# Neuron-glia interaction through Serotonin-BDNF-NGFR axis enables regenerative neurogenesis in Alzheimer’s model of adult zebrafish brain

**DOI:** 10.1371/journal.pbio.3000585

**Published:** 2020-01-06

**Authors:** Prabesh Bhattarai, Mehmet Ilyas Cosacak, Violeta Mashkaryan, Sevgican Demir, Stanislava Dimitrova Popova, Nambirajan Govindarajan, Kerstin Brandt, Yixin Zhang, Weipang Chang, Konstantinos Ampatzis, Caghan Kizil

**Affiliations:** 1 German Center for Neurodegenerative Diseases (DZNE) within Helmholtz Association, Dresden, Germany; 2 CRTD—Center for Regenerative Therapies Technische Universität Dresden, Dresden, Germany; 3 B CUBE, Center for Molecular Bioengineering, Technische Universität Dresden, Dresden, Germany; 4 Karolinska Institutet, Neuroscience Department, Stockholm, Sweden; UCSD, UNITED STATES

## Abstract

It was recently suggested that supplying the brain with new neurons could counteract Alzheimer’s disease (AD). This provocative idea requires further testing in experimental models in which the molecular basis of disease-induced neuronal regeneration could be investigated. We previously found that zebrafish stimulates neural stem cell (NSC) plasticity and neurogenesis in AD and could help to understand the mechanisms to be harnessed for developing new neurons in diseased mammalian brains. Here, by performing single-cell transcriptomics, we found that amyloid toxicity-induced interleukin-4 (IL4) promotes NSC proliferation and neurogenesis by suppressing the tryptophan metabolism and reducing the production of serotonin. NSC proliferation was suppressed by serotonin via down-regulation of brain-derived neurotrophic factor (BDNF)-expression in serotonin-responsive periventricular neurons. BDNF enhances NSC plasticity and neurogenesis via nerve growth factor receptor A (NGFRA)/ nuclear factor 'kappa-light-chain-enhancer' of activated B-cells (NFkB) signaling in zebrafish but not in rodents. Collectively, our results suggest a complex neuron-glia interaction that regulates regenerative neurogenesis after AD conditions in zebrafish.

## Abstract 2

Can regeneration of lost neurons counteract neurodegenerative disease? This study shows that serotonergic neurons alter neural stem cell proliferation and neurogenesis via a complex neuron-glia interaction involving interleukin-4, BDNF and NGF receptor in a zebrafish model of Alzheimer's disease.

## Introduction

Alzheimer’s disease (AD) entails versatile pathological changes such as synaptic degeneration, neuronal death, chronic inflammation, impaired vasculature function, and reduced plasticity of neural stem cells (NSCs) [1–3]. The cognitive decline that is observed in AD patients and experimental animal models is mainly caused by the reduced neural network integrity [4]. The efforts to rescue the cognitive decline and neuronal death traditionally largely focused on the neuronal compartment and aimed at preventing the death of the neurons, but this has not yielded in desired success in clinics [5,6]. An alternative approach was suggested to complement the treatment in neuronal compartments by increasing the production of new neurons to provide resilience and strength to the diseased circuitry [3,7–9]. Yet, neurogenesis in human brains is rather controversial [10–14]. Although many reports documented the presence of adult neurogenesis in humans [3,15–19], and several studies demonstrated that boosting the neurogenesis might be a viable option for alleviating the cognitive decline [7,20–23], the potential benefits of neurogenic outcome in AD conditions requires further investigation and critical testing. Additionally, in AD conditions, mammalian NSCs reduce the proliferative ability dramatically, and for neurogenesis to become a viable option for treatment of neurological disorders, mammalian NSCs must become plastic first. Therefore, examining how NSCs could be made proliferative and neurogenic during the course of AD could provide important clinical ramifications toward the treatment of this disease [24–26]; however, little is known about the mechanisms by which neural stem cells would enhance their proliferative response [16,27,28]. Recently, we established a zebrafish model, which can recapitulate the symptoms of AD in humans, such as neuronal death, synaptic degeneration, chronic inflammation, and cognitive decline [29–32]. Interestingly, in contrast to humans, the zebrafish brain could enhance NSC proliferation and neurogenesis through a previously unidentified neuro-immune regulation involving Interleukin-4 (IL4). IL4 secreted by dying neurons activated microglia, which in turn activated NSC proliferation [30]. We also found that IL4 could revert the pathological effects on NSC in an in vitro 3D reductionist model of AD [20]. Yet, the mechanisms by which IL4 regulates NSC proliferation and neurogenesis after amyloid toxicity remained unknown. In this manuscript, using single-cell sequencing, we identified a previously undocumented IL4-dependent mechanism that regulates NSC plasticity in the adult zebrafish brain, by which IL4 regulates production of serotonin, which suppresses production of the brain-derived neurotrophic factor (BDNF) in periventricular neurons juxtaposing the NSCs. We found that BDNF is required to activate NSC plasticity, proliferation of NSCs, and neurogenesis through its receptor nerve growth factor receptor A (NGFRA), the blockage of which reduces NSC proliferation. Overall, our results identify a mechanism by which IL4 regulates NSC plasticity through serotonin-dependent expression of BDNF in neurons in the zebrafish brain and demonstrate functional heterogeneity of NSCs based on receptor expression. Our results will provide a conceptual basis for neuro-immune regulation of NSCs, neuronal control of NSC proliferation, and differential response of NSC subtypes to various signals in zebrafish. Such understanding could be instrumental in the efforts to develop novel therapies for AD through increased neurogenesis.

## Results

**Fig 1:**
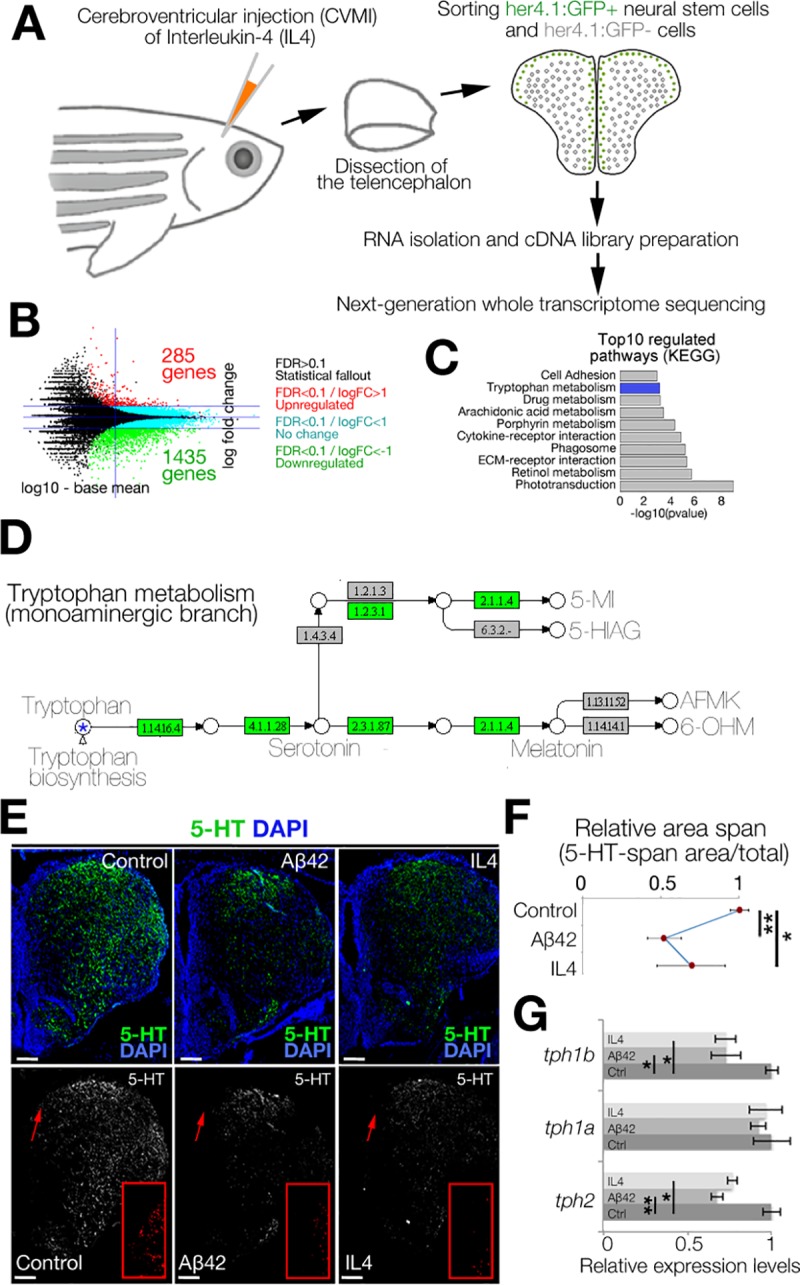
IL4 regulates tryptophan metabolism. (A) Schematic view of the experimental pipeline for whole-transcriptome sequencing IL4 treatment. (B) MA-plot for DEGs. (C) GO-term analyses on DEGs. (D) Modified KEGG-pathway view of tryptophan metabolism. Green indicates the enzymes down-regulated by IL4. (E) IHC for 5-HT in control (left), Aβ42-injected (middle), and IL4-injected (right) brains. Single-channel images show 5-HT. Red insets are high-magnification images of arrowed regions. (Dm: dorsal-medial) (F) Quantification of 5-HT-span area density under the conditions of E. (G) qRT-PCR results for *tph1a*, *tph1b*, and *tph2* in control, amyloid-injected, and IL4-injected zebrafish brains. Beta-actin used for normalization. *n =* 3 animals for experiments. Scale bars equal 100 μM. Data are represented as mean ± SEM. See also S1 Fig and S2 Fig. See S2 Data and S7 Data for supporting information. Aβ42, amyloid-beta42; DEG, differentially expressed gene; ECM, extracellular matrix; FC, fold change; FDR, false discovery rate; GFP, green fluorescent protein; GO, gene ontology; IHC, immunohistochemistry; KEGG, Kyoto Encyclopedia of Genes and Genomes; IL4, interleukin-4; MA, Bland-Altman mean-average plot; qRT-PCR, quantitative real-time polymerase chain reaction; 5-HT, serotonin.

To determine the effects of IL4 on NSC plasticity in the adult zebrafish brain, we administered IL4 through cerebroventricular microinjection, dissected the telencephalon at 1 day post injection (dpi), separated the her4.1: green fluorescent protein (GFP)-positive NSCs from GFP-negative cells, and performed whole-transcriptome profiling in control and IL4-injected brains for both of these cell populations (Fig 1A, S1 Data). When we compared the gene expression profiles in GFP-negative cell populations that contain non-NSC cell types, including the neurons and immune cells, we found that IL4 administration increased the expression of 285 genes and down-regulated 1,435 genes (Fig 1B, S1 Data). To determine the pathways affected by IL4, we performed Kyoto Encyclopedia of Genes and Genomes (KEGG) analyses and observed that one of the significantly regulated pathways was tryptophan metabolism (Fig 1C, S1 Fig). Specifically, we observed that the enzymes generating serotonin from tryptophan were down-regulated (Fig 1D, S2 Data), suggesting that IL4 might reduce the production of serotonin (5-HT). To test whether amyloid-beta42 (Aβ42, which induces IL4 expression [30]) and ectopic IL4 would reduce the availability of 5-HT in adult zebrafish brain, we performed immunohistochemistry for 5-HT (Fig 1E) and observed that Aβ42 and IL4 significantly reduces the 5-HT immunoreactivity at 1 dpi in adult zebrafish telencephalon (Fig 1F), and this is due to reduced expression of enzyme *tph2*, an enzyme responsible for production of serotonin (Fig 1G). We found that Aβ42 or IL4 does not induce the death of 5-HT cells because the cell bodies of serotonergic cells present in superior raphe region are intact and are not, terminal deoxynucleotidyl transferase dUTP nick end labeling (TUNEL)-positive after Aβ42 or IL4 treatment (S2 Fig). These results demonstrate that Aβ42 and IL4 suppress serotonin production rather than the innervation. Additionally, the effects of IL4 is specific to serotonergic system, because in our deep sequencing analyses we did not observe changes in KEGG analyses in other neurotransmitter pathways, such as dopamine, histamine, or noradrenalin (S2 Data).

**Fig 2:**
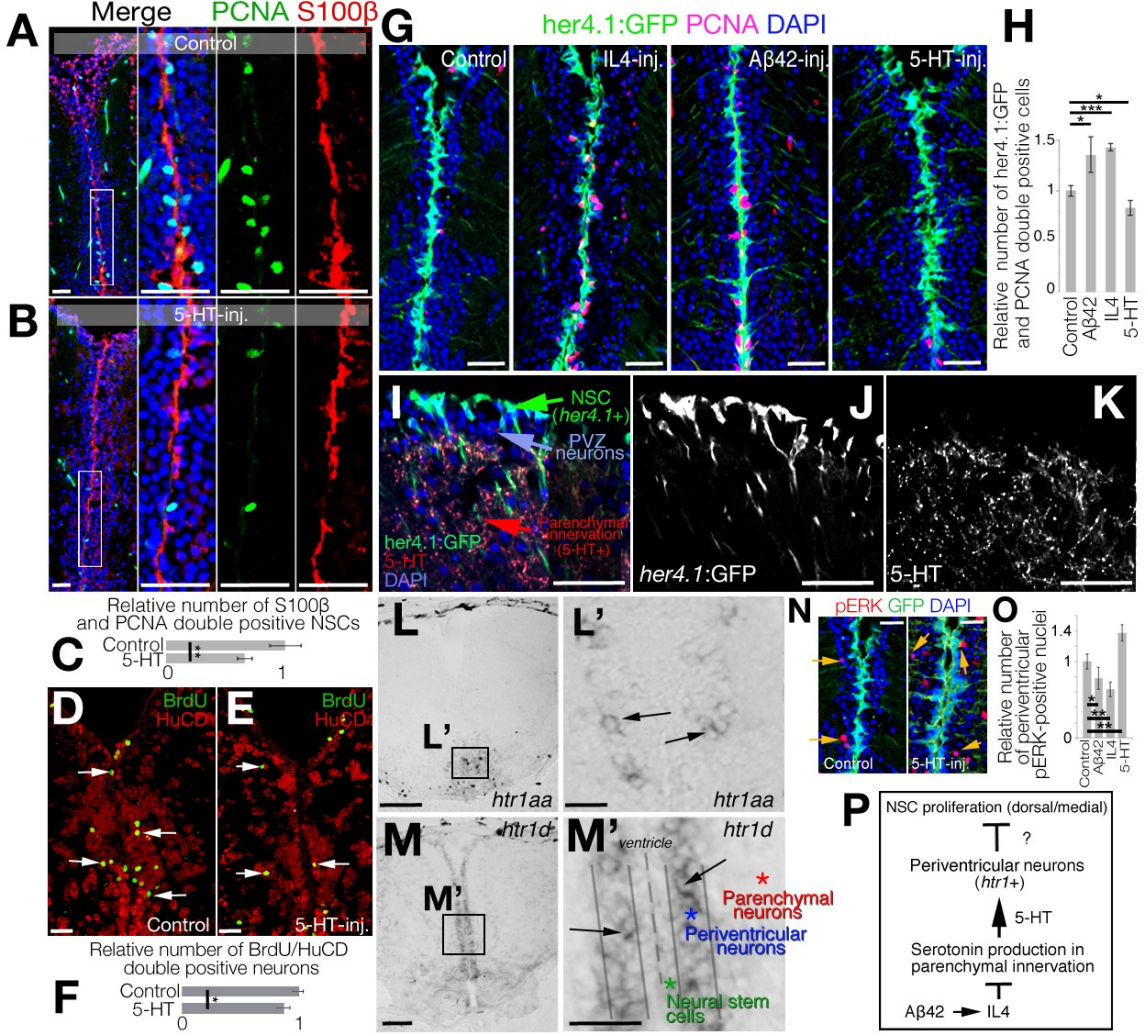
Serotonin regulates NSC plasticity indirectly through HTR1 signaling in periventiruclar neurons. (A, B) IHC for S100β and PCNA in control (A) and 5-HT-injected (B) brains. (C) Quantification of proliferating glia in conditions of panels A and B. (D, E) IHC for BrdU and HuC/D for newborn neurons at 14 dpi after BrdU treatment at 2 and 3 dpi (D) and BDNF injection (E). (F) Quantification of newborn neurons. (G) IHC for GFP (driven by glial promoter her4.1) and PCNA in control, IL4-injected, Aβ42-injected, and 5-HT-injected brains. (H) Quantification of proliferating glia in conditions of panel G. (I-K) IHC for her4.1-driven GFP and 5-HT. The composite image (I) and single fluorescent channels for her4.1:GFP (J) and 5-HT (K). (L) ISH for *htr1a* (panel Lʹ: close-up image of framed region in panel L). (M) ISH for *htr1d* (panel Mʹ: close-up image of framed region in panel M). (N) IHC for pERK and her4.1-driven GFP in control and 5-HT-injected brains. (O) Quantification of pERK-positive periventricular neurons. (P) Working hypothesis on the indirect regulation of 5-HT on NSC plasticity. *n =* 4 animals for experiments. Scale bars equal 100 μM. Data are represented as mean ± SEM. See also S3 Fig. See S7 Data for supporting information. Aβ42, amyloid-beta42; BDNF, brain-derived neurotrophic factor; BrdU, 5-bromo-2'-deoxyuridine; dpi, days post injection; GFP, green fluorescent protein; HTR1, 5-hydroxytryptamine receptor 1; HuC/D, ELAV (embryonic lethal, abnormal vision, Drosophila)-like 3/4; IHC, immunohistochemistry; IL4, interleukin-4; ISH, in situ hybridization; NSC, neural stem cell; pERK,; phosphorylated extracellular signal-regulated kinase; PCNA, proliferating cell nuclear antigen; PVZ, periventricular zone; S100β, S100 calcium-binding protein B; 5-HT, serotonin.

Because Aβ42/IL4 enhances NSC proliferation in the adult zebrafish brain [29,30,32,33] and they down-regulate 5-HT (Fig 1E and 1F), we hypothesized that 5-HT could be negatively affecting NSC plasticity. To test this, we injected 5-HT into the adult zebrafish brain and analyzed the proliferation of NSCs (S100 calcium-binding protein B (S100β) and and proliferating cell nuclear antigen (PCNA) double positive cells) at 1 dpi (Fig 2A–2C). We observed a significant reduction (reduced by 32%) of NSC proliferation after 5-HT injection (Fig 2C). This reduction has implications in neurogenesis from NSCs, because the analysis of 5-bromo-2'-deoxyuridine (BrdU)-labeled newborn neurons at 14 dpi of PBS (Fig 2D) or 5-HT (Fig 2E) showed that 5-HT reduces the neurogenic outcome by 12% (Fig 2F). To verify our results, we used a transgenic reporter line that marks NSCs with GFP driven under the *her4*.*1* promoter and injected Aβ42, IL4, or 5-HT followed by immunohistochemistry (IHC) for GFP and PCNA at 1 dpi (Fig 2G). Consistent with our previous results, Aβ42 and IL4 injection increased NSC proliferation by 35% and 42%, respectively, whereas 5-HT reduced the number of proliferating progenitors by 18% (Fig 2H), indicating that 5-HT has a negative impact on NSC plasticity in zebrafish telencephalon. To further test whether the negative effects of 5-HT would be reversed by Aβ42 or IL4, we co-injected 5-HT with Aβ42 and IL4 and observed that the reduction in NSC proliferation by 5-HT can be abrogated by Aβ42 or IL4 (S3 Fig), indicating that 5-HT signaling acts antagonistically to IL4 in NSCs.

Our previous results indicated that a subset of NSCs express IL4 receptor and can therefore be directly regulated by IL4 [24,30,34]. Therefore, we aimed to investigate whether 5-HT would affect the same subset of NSCs or whether it affects a distinct population. To determine whether or not 5-HT has a direct effect on NSCs, we determined the spatial organization of NSCs (*her4*.*1*:GFP, Fig 2I and 2J) and 5-HT innervation (Fig 2I and 2K). We observed that NSCs are located apically and are separated by periventricular zone (PVZ) neurons before the proximal front of the serotonergic innervation in the parenchyma (Fig 2I), suggesting that the effect of 5-HT on NSCs could be indirect. If this hypothesis was true, NSCs would not express 5-HT receptors. To determine which cells expressed 5-HT receptors, we performed in situ hybridization (ISH) for 5-HT receptors (*htr* genes) and observed that among 7 serotonin receptor genes and in total 21 isoforms of those genes (S2 Data), only *htr1a* and *htr1d* gave ISH signals in adult zebrafish telencephalon (Fig 2L–2Mʹ). We observed that serotonin receptor genes are not expressed in the progenitor cells (S3 Fig), supporting previous findings [35] and suggesting that serotonin does not act directly on NSCs. To further investigate this hypothesis, we performed electrophysiological recordings from 9 her4.1:GFP-positive NSCs and 7 adjacent her4.1:GFP-negative cells (neurons; S3 Fig). We found that out of 9 recorded NSCs (3 of them in the presence of tetrodotoxin [TTX]), none responded to serotonin (S3 Fig). For the neurons, we recorded 8 (2 in the presence of TTX), and 7 of them responded to serotonin by increasing the frequency of the recorded excitatory postsynaptic currents (EPSCs) (S3H Fig). This further confirms that serotonin is indeed affecting periventricular neurons but not NSCs in the adult zebrafish brain.

Among the serotonin receptor genes, *htr1a* was expressed only in ventral regions (Fig 2L and 2Lʹ), whereas *htr1d* was present in periventricular region immediately adjacent to the ventricular zone containing the NSCs that span the medial and dorsal regions of the telencephalon (Fig 2M and 2Mʹ). These findings suggested that the effect of 5-HT on NSCs is not direct, and it could be mediated through periventricular neurons expressing *htr1d* (Fig 2N). If 5-HT would act on periventricular neurons, 5-HT would activate its downstream effector phosphorylated extracellular signal-regulated kinase (pERK) [36] in periventricular regions. Indeed, we found that compared with controls in which nuclear pERK is in few cells at the periventricular zone (PVZ) (Fig 2O), 5-HT injection increases the number of pERK+ nuclei (by 37%) only in the PVZ region (Fig 2N). Quantification of pERK+ nuclei also confirmed that 5-HT increases pERK+ nuclei on the contrary to Aβ42/IL4 (Fig 2O), indicating that 5-HT directly affects periventricular neurons but not the NSCs (Fig 2P).

To determine the mechanistic link between 5-HT and NSC plasticity through *hrt1*+ cells (Fig 2P), we performed single-cell transcriptomics in control and 5-HT-treated adult zebrafish telencephalon by unbiased clustering, determination of cell types and differential gene expression analyses using the methodology we have recently developed [34] (S4A Fig; S3 Data). After quality control analyses (S4B Fig), we obtained t-distributed stochastic neighbor embedding (tSNE) clusters, which dissolve into 4 major cell types: neurons (*eno2*, *gap43*, *map1aa*, *sypb*-positive), glia (*gfap*, *her4*.*1*, *msi1*, *s100b*-positive), oligodendrocytes (*aplnrb*, *olig2*-positive), and immune cells (*pfn*, *lcp1*-positive; S4C–S4E Fig). Heat maps reveal marker genes expressed predominantly in those cells (S4D Fig). To determine the cells that are responsive to 5-HT, we plotted the cells expressing *htr1* and observed that *htr1* expression was confined to neurons (S4F Fig). This expression pattern was consistent with ISH results of *htr1 genes* (Fig 2M, S3G Fig) and was not overlapping with *il4r*.*1-*expressing cells that were exclusively glia and immune cells ([30] and S4F Fig).

**Fig 3:**
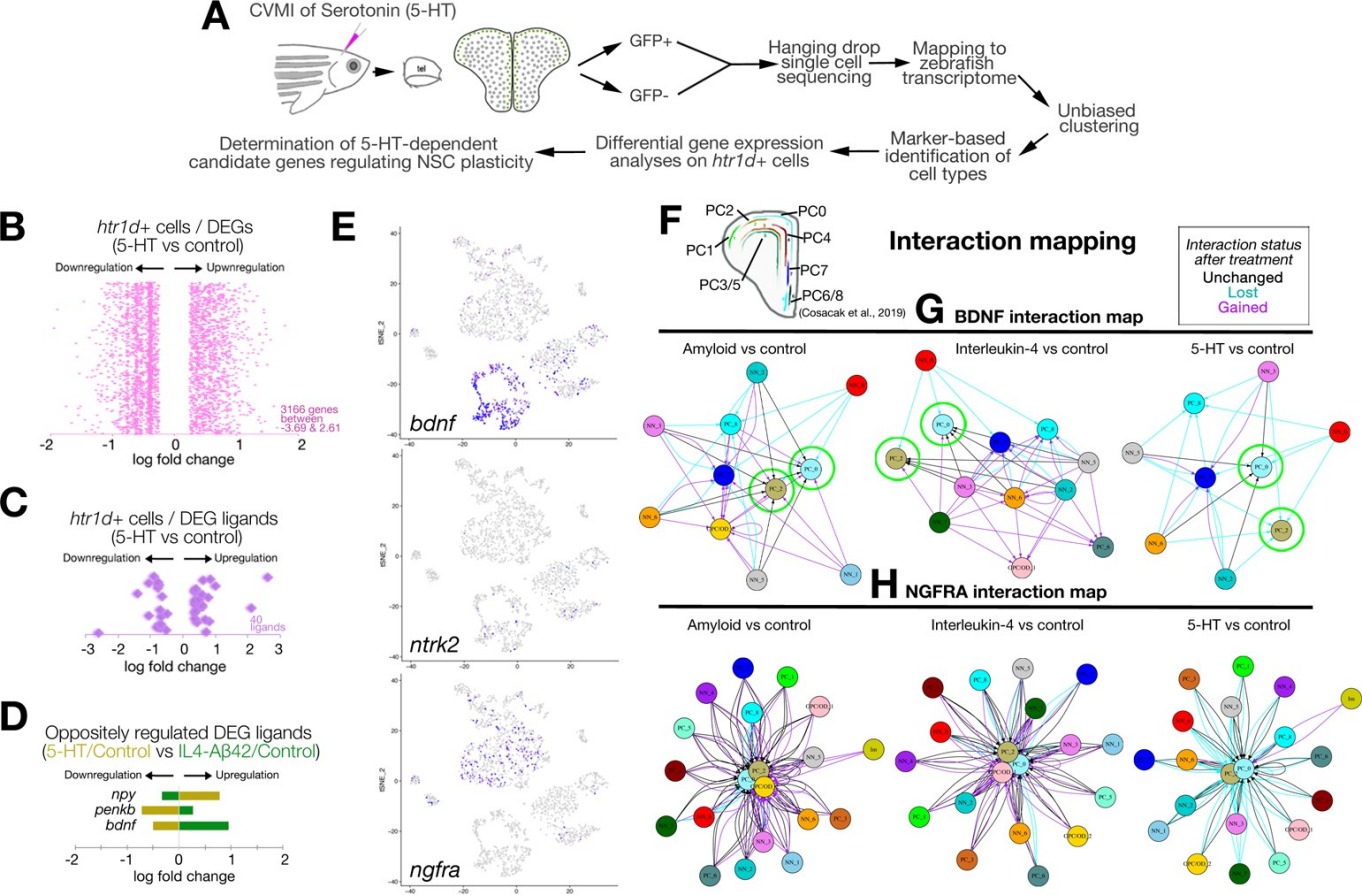
Single-cell sequencing after serotonin treatment in the adult zebrafish brain and data analyses. (A) Schematic workflow for single-cell sequencing and data analyses. (B) Distribution plot for DEGs in *htr1d*-expressing cells after 5-HT treatment. (C) Ligands selected from panel B. (D) Plots for ligands that change oppositely in 5-HT and IL4/Aβ42 treatment. (E) Feature plots for *bdnf* and its receptors *ntrk2* and *ngfra*. (F) Spatial map of NSCs/PCs in the adult zebrafish brain as previously described in Cosacak et al., 2019. (G) In silico interaction map for BDNF in amyloid versus control, IL4 versus control, and 5-HT versus control comparisons. (H) In silico interaction map for NGFRA in Aβ42 versus control, IL4 versus control, and 5-HT versus control comparisons. In panels G and H, black arrows: interactions unchanged with treatment, cyan arrows: interaction lost with treatment, magenta arrows: interaction gained/emerged with the treatment. See also S4 Fig and S5 Fig. See S3 Data and S4 Data for supporting information. Aβ42, amyloid-beta42; BDNF, brain-derived neurotrophic factor; CVMI, cerebroventricular microinjection; DEG, differentially expressed gene; GFP, green fluorescent protein; IL4, interleukin-4; NGFRA, nerve growth factor receptor A; NSC, neural stem cell; PC, progenitor cell; 5-HT, serotonin.

To determine the mechanism by which 5-HT-responsive cells affect glia proliferation, we devised an analysis pipeline in which we dissected the *htr1d*+ cells from the rest and performed differential expression analysis between control and 5-HT-treated brains (Fig 3A; S4G Fig). We found that 3,166 genes changed their expression levels in *htr1d*+ cells after 5-HT treatment (Fig 3B, S4 Data). We hypothesized that a possible regulation between *htr1d*+ neurons and NSCs could be through a paracrine ligand–receptor crosstalk. We found that in *htr1d*+ cells, 40 ligands change their expression levels (Fig 3C), 3 of which also change their expression reciprocally after 5-HT and IL4/ Aβ42 treatment (Fig 3D, and data from [34]). The highest up-regulation was observed for *bdnf* (Fig 3D). From single-cell analyses, we found that *bdnf* was predominantly expressed in neuronal clusters (90% in neurons and 8% in glial cells; Fig 3E, S4H Fig), whereas its receptor *ntrk2* [37,38] was expressed mainly in neurons (68% neuronal and 28% glia; Fig 3E, S4H Fig), and another BDNF receptor *ngfra* [37–39] was mostly glial (93% in glia and 5% in neurons; Fig 3E; S4H Fig). Therefore, we hypothesized that 5-HT dependent regulation of *bdnf* expression might signal to the glial cells through *ntrk2* and *ngfra* in the adult zebrafish brain.

To further investigate whether BDNF signaling through Ntrk2 or Ngfra would affect NSCs, we used an in silico interaction map analysis that we recently developed [34]. According to this analysis, if BDNF had a potential interaction between neuronal and glial (progenitor) clusters, we would see an in silico interaction between these cells. Alternatively, if *ntrk2* or *ngfra* could constitute a crosstalk between neuronal and glial clusters, we would be able to see an interaction (see Cosacak and colleagues [34] for details of interaction mapping). For interaction analyses, we used the spatial organization map of adult zebrafish NSCs in the telencephalon (Fig 3F), distinguished the cell types based on our previous findings (Cosacak and colleagues [34]) by using machine learning algorithms, and compared the 3 treatments with controls (IL4 versus control, amyloid versus control, and 5-HT versus control; Fig 3G and 3H; S5 Fig). For mapping, we used the following 3 criteria: (1) we took either the *bdnf* as a starting point (i.e., the cells expressing *bdnf* are matched with the cells expressing any *bdnf* receptor, or the *bdnf* receptors *ntrk2* and *ngfra* were taken as starting points [i.e., only *ntrk2* or *ngfra*-expressing cells were matched with cells expressing any ligand for those receptors (Fig 3H for *ngfra* and S6A Fig for *ntrk2*)]); (2) charted the potential interactions as arrows starting from *bdnf*-expressing clusters to *bdnf* receptor-expressing clusters (Fig 3G) or to *bdnf* receptor-expressing clusters from clusters expressing their ligands (Fig 3H), and (3) depending on the change of the interaction after any treatment, we color-coded the interactions (black arrows indicate interactions that are unchanged by the treatment, cyan arrows indicate the interactions that are lost after treatment, and magenta arrows indicate interactions that emerged after a particular treatment). After these analyses, we found that especially 2 clusters of progenitor cells (PC0 and PC2, which are located to dorsal and medial region of the telencephalon; Fig 3F and Cosacak and colleagues [34]) were among the clusters that had the highest number of arrows pointing toward them (Fig 3G). Interestingly, the majority of the potential interactions were newly generated after amyloid or IL4 treatment, whereas those interactions are mainly lost after 5-HT treatment (Fig 3G, green circles), suggesting that the regulation of progenitor cells (NSCs) by *bdnf* could be promoted by amyloid or IL4 treatment and suppressed by 5-HT, which is consistent with our hypothesis. Additionally, to support our findings, we performed independent mapping analyses of *bdnf* receptors *ntrk2* and *ngfra*. We constructed interaction maps for these receptors and found that *ngfra* might be the main receptor for *bdnf* signaling in adult zebrafish telencephalon because 2 NSC clusters (PC0 and PC2) were at the center of the interaction maps with many potential interactions (Fig 3H, S4 Fig), whereas the *ntrk2* interaction map provided only a small number of interactions to another glial cluster (S4 Fig, S6A Fig). We observed that IL4 activates many interaction routes to NSC clusters whereas 5-HT almost diminishes all the interactions between NSCs and other cells (Fig 3H). These in silico analyses suggest that IL4 promotes the interactions between neurons and NSCs whereas 5-HT suppresses such interactions.

**Fig 4:**
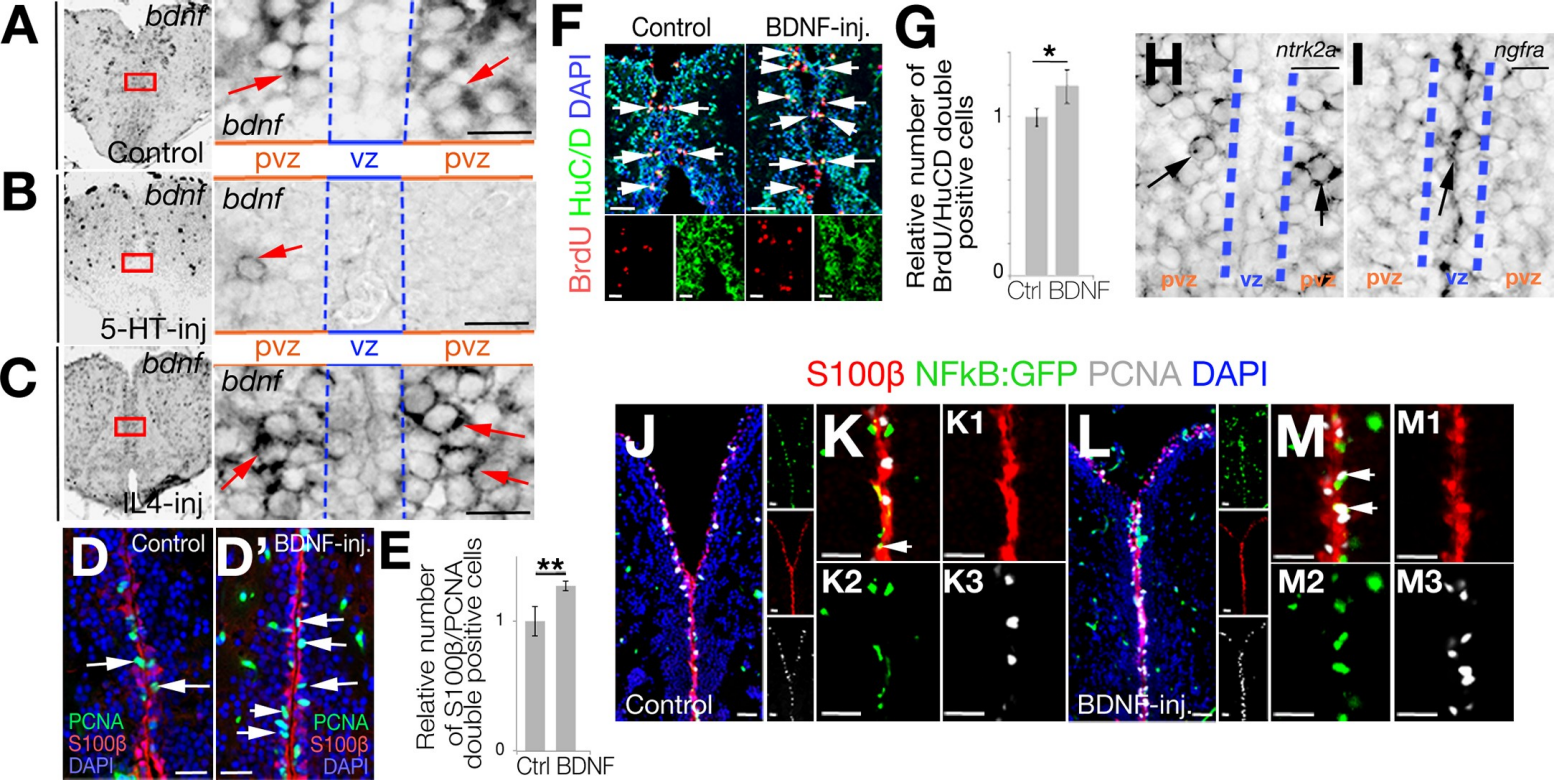
Serotonin regulates periventricular *bdnf* expression and NFkB signaling in NSCs. (A–C) ISH for *bdnf* in control (A), 5-HT-injected (B), and IL4-injected (C) brains. Red rectangles are enlarged to the right of the main panels. Note significant reduction of *bdnf* expression after 5-HT. (D, Dʹ) IHC for PCNA and S100β in control (D) and BDNF-injected (Dʹ) brains. (E) Quantification of proliferating glial cells after BDNF injection. (F) IHC for BrdU and HuC/D in control and BDNF-injected brains. (G) Quantification of newborn neurons at 14 dpi after BrdU treatment at Day 2 and Day 3 after PBS and BDNF injection. (H) ISH for *ntrk2*, which is expressed in periventricular neurons but not in NSCs in the vz. (I) ISH for *ngfra*, which is expressed in vz. (J) IHC for S100β, NFkB-driven GFP, and PCNA in control brains. To the right of the larger panel are single-fluorescence channels. (K) High-magnification of the medial region of panel J without DAPI. (K1–K3) Single fluorescent channels of panel K. (L) IHC for S100β, NFkB-driven GFP, and PCNA in BDNF-injected brains. To the right of the larger panel are single-fluorescence channels. (M) High-magnification of the medial region of panel L without DAPI. (M1–M3) Single-fluorescence channels of panel M. Scale bars equal 100 μM. Data are represented as mean ± SEM. See also S4–S10 Figs. See S7 Data for supporting information. BDNF, brain-derived neurotrophic factor; BrdU, 5-bromo-2'-deoxyuridine; dpi, days post injection; GFP, green fluorescent protein; HuC/D, ELAV (embryonic lethal, abnormal vision, Drosophila)-like 3/4; IHC, immunohistochemistry; IL4, interleukin-4; ISH, in situ hybridization; NFkB, nuclear factor 'kappa-light-chain-enhancer' of activated B-cells; NSC, neural stem cell; PCNA, proliferating nuclear cell antigen; pvz, periventricular zone; S100β, S100 calcium-binding protein B; vz, ventricular zone; 5-HT, serotonin.

To verify our in silico analyses for interaction mapping on single-cell transcriptomics data and to investigate the changes in the expression of *bdnf*, we performed ISH on control (Fig 4A), 5-HT-injected (Fig 4B), and IL4-injected brains (Fig 4C). The expression of *bdnf* in control brains was in the PVZ proximal to the NSCs, which confirmed our single-cell transcriptomics data. The expression of *bdnf* was almost abolished by 5-HT injection (Fig 4B), whereas it is enhanced by IL4 (Fig 4C), suggesting that 5-HT suppresses NSC plasticity through reducing the *bdnf* signaling.

Given that 5-HT suppresses *bdnf* expression and NSC proliferation whereas IL4 enhances *bdnf* expression and NSC proliferation, we hypothesized that *bdnf* would also enhance NSC plasticity by increasing cell proliferation. To test this, we injected BDNF into adult zebrafish brains (Fig 4D and 4E) and observed that BDNF indeed increases the number of proliferating NSCs by 27% at 1 dpi (Fig 4E). The enhanced proliferation of NSCs by BDNF is also translated into increased neurogenesis because BDNF-injected brains produce more neurons (increase by 19%) compared with control-injected zebrafish brains (Fig 4F and 4G), indicating that BDNF enhances NSC plasticity in the adult zebrafish brain.

If BDNF would act directly on NSCs, its receptor must have been present in the target cells. Therefore, we performed ISH for *ntrk2* and *ngfra* in adult zebrafish brains. We observed that *ntrk2* mRNA is expressed mainly in the periventricular region of the telencephalon (Fig 4H and S6B Fig), which is supported by IHC staining for NTRK2 protein (S6C Fig). On the contrary, *ngfra* is expressed mainly in the ventricular zone where NSCs reside (Fig 4I, S6 Fig). These findings are perfectly matching with our single-cell transcriptomics data (Fig 3E, S4H Fig) and suggest that *ngfra* is the primary receptor for *bdnf* in NSCs and *ntrk2* is the primary receptor in neurons. To test this hypothesis, we injected BDNF into the adult zebrafish brain and determined the downstream effectors of NTRK2 and NGFRA signaling pathways. To determine the cells responding to BDNF/NTRK2 signaling, we detected the downstream effector phosphorylated protein kinase B (pAkt) [36] and observed that after BDNF injection, pAkt is almost exclusively present in periventricular cells and parenchymal neurons (few speckles in ventricular region constitute less than 0.1% of the pAkt signal when compared with the intensity and number of cells outside the ventricular region; S6D and S6E Fig). On the contrary, compared with control-injected brains, BDNF injection increased the activity of the NFkB reporter [40], which is the downstream effector of NGFR signaling [41] in the ventricular region where NSCs reside (Fig 4J–4M3). Additionally, consistent with our previous result, we observed that at 1 dpi, NFkB signaling and the number of NFkB-positive proliferating NSCs (S100β /PCNA/NFkB:GFP-triple positive cells) increased by 47% after BDNF injection, 28% after Aβ42 injection, and 25% after IL4 injection but reduced by 10% after 5-HT injection (S6 Fig). These results indicate that NGFR-mediated intracellular signaling is the primary route for the 5-HT-dependent BDNF activity on NSCs in the adult zebrafish brain.

**Fig 5:**
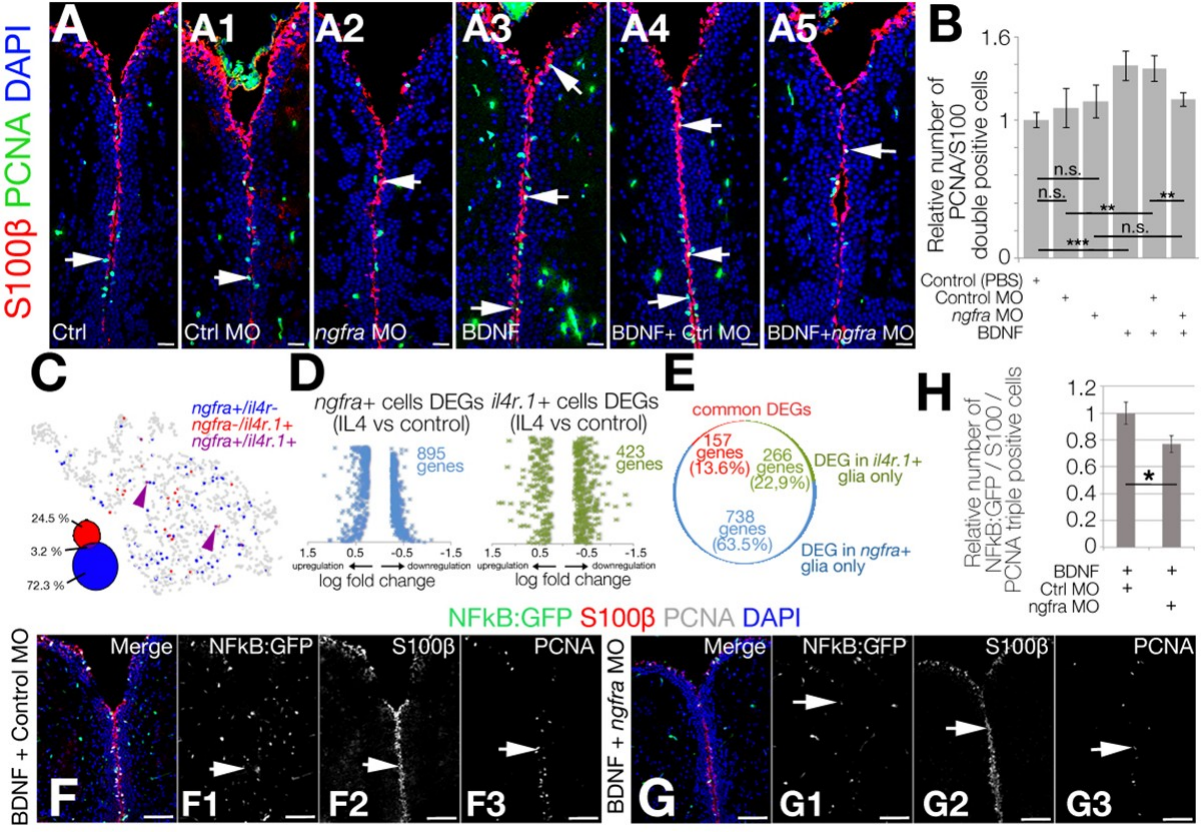
Ngfra signaling regulates NSC plasticity independent of Il4ra signaling. (A–A5) IHC for S100β and PCNA in control (A), control morpholino-injected (A1), *ngfra* morpholino-injected (A2), BDNF-injected (A3), BDNF + control morpholino-injected (A4), and BDNF + *ngfra* morpholino-injected (A5) brains. (B) Quantification for the relative number of proliferating glial cells. (C) Co-representation of *ngfra* and *ilr4* expressions on glial tSNE plot. (D) DEG plots for *ngfra-*positive and *il4r*-positive neural stem cells after IL4 treatment. (E) Pie chart distribution of unique DEGs. Note that the overlapping genes constitute only 13.6% of all DEGs. (F, G) IHC for S100β, NFkB-driven GFP, and PCNA in BDNF + control morpholino-injected (F–F3) and BDNF + *ngfra* morpholino-injected brains (G–G3).(H) Quantification graph for relative numbers of proliferating stem cells with active NFkB signaling. Scale bars equal 100 μM. Data are represented as mean ± SEM. See also S10 Fig. See S5 Data, S6 Data, and S7 Data for supporting information. BDNF, brain-derived neurotrophic factor; DEG, differentially expressed gene; GFP, green fluorescent protein; IHC, immunohistochemistry; IL4, interleukin-4; NFkB, nuclear factor 'kappa-light-chain-enhancer' of activated B-cells; Ngfr, nerve growth factor receptor; NSC, neural stem cell; PCNA, proliferating nuclear cell antigen; S100β, S100 calcium-binding protein B; tSNE, t-Distributed Stochastic Neighbor Embedding.

If our hypothesis was true and BDNF would regulate NSC proliferation through NGFRA and NFkB signaling, knocking-down the *ngfra* receptor after BDNF injection would suppress the increase in NSC proliferation by BDNF as well as the increase in NFkB signaling. To test this hypothesis, we knocked down *ngfra* by using morpholino oligonucleotides and determined the extent of proliferating NSCs (Fig 5A–5A5). Compared with control brains, BDNF increased the NSC proliferation by 38%; control morpholino did not alter this increase (remained 37% increase) whereas *ngfra* morpholino diminished (reduced down to 13%) the increased NSC proliferation upon BDNF injection (Fig 5B).

**Fig 6:**
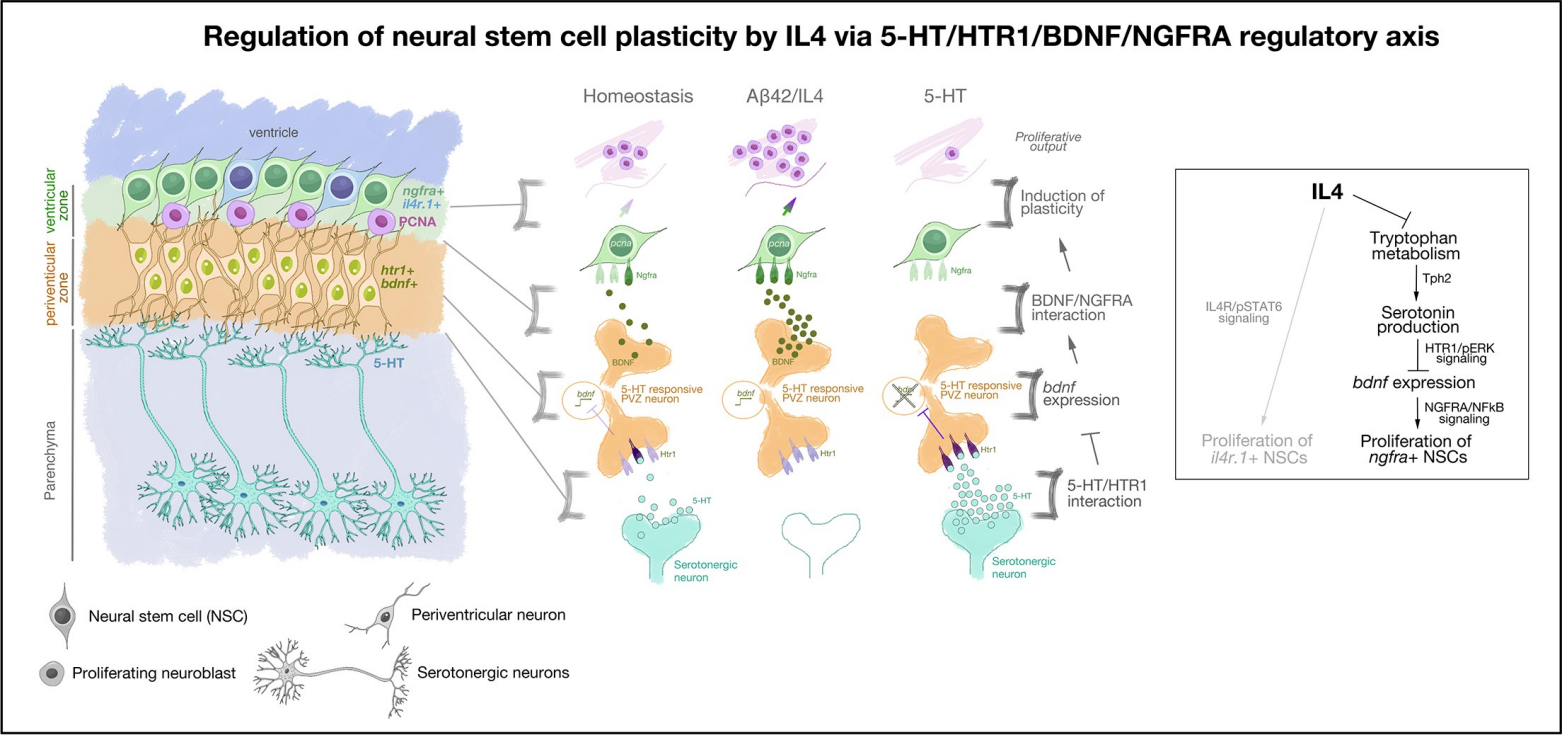
Schematic view of the findings on how neuron-glia crosstalk regulates Alzheimer-induced neurogenesis in adult zebrafish brain. Aβ42, amyloid-beta42; bdnf, brain-derived neurotrophic factor; htr1, 5-hydroxytryptamine receptor 1; IL4, interleukin-4; NFkB, nuclear factor 'kappa-light-chain-enhancer' of activated B-cells; NGFRA, nerve growth factor receptor A; NSC, neural stem cell; PCNA, proliferating cell nuclear antigen; pERK, hosphorylated extracellular signal-regulated kinase; pSTAT6, phosphorylated signal transducer and activator of transcription 6, interleukin-4 induced; PVZ, periventricular zone; ngfra, nerve growth factor receptor A; Tph2, tryptophan hydroxylase; 5-HT, serotonin.

Because BDNF/NGFRA signaling is affected by IL4 that also acts directly on NSCs through IL4R [30], we hypothesized that the effect of IL4 on NSCs could be mediated through IL4R (directly by IL4/IL4R interaction) and NGFR (through 5-HT/BDNF/NGFRA/NFkB axis) distinctly, and IL4R-positive and NGFRA-positive glia would constitute 2 functional subtypes of NSCs. To determine whether IL4R-positive and NGFRA-positive NSCs are distinct subtypes of NSCs in adult zebrafish brain, we plotted both cell populations on the same tSNE plot (Fig 5C). We observed that only 3.2% of *ngfra-*positive NSCs were also *il4r1-*positive (24.5% are only *il4r*-positive and 72.3% are only *ngfra*-positive), indicating that these 2 populations are likely to represent 2 functional subtypes of NSCs. We further hypothesized that if *ngfra*-positive and *il4r*-positive NSCs would constitute different subtypes, their response to particular treatments would also lead to distinct differential gene expression profiles. To address this question, we determined the overlap between differentially expressed genes in *il4r*-positive and *ngfra*-positive NSCs (Fig 5D). We determined that, after IL4 treatment, 423 and 895 genes are differentially expressed in *il4r*+ (S5 Data) and *ngfra*+ (S6 Data) NSCs, respectively (Fig 5D). Only 14% of the DEGs are common in these cell populations whereas the rest are differentially expressed in only one of the cell types (Fig 5E). Similarly, after BDNF treatment, *ngfra* knockdown reduced the number of proliferating NSCs where NFkB signaling is active (Fig 5F–5H). These results indicate that neuron-glia interaction through BDNF regulates NSC proliferation and neurogenesis through NGFRA/NFkB signaling (Fig 6).

To explore the evolutionary conservation of our findings in healthy and AD conditions, we determined the expression of BDNF, NTRK2, and p75/NTR (NGFRA ortholog in mouse) in wild-type mouse and APP/PS1dE9 AD model (S7–S9 Figs). Compared with 12-month-old control mouse brains, age-matched APP/PS1dE9 mouse displayed reduced SOX2 (neurogenic competency and NSC maintenance marker [42,43]) and increased GFAP (glial marker [44,45]; S7 Fig, S8 Fig, S9 Fig) that is indicative of reduced neurogenic ability and increased gliosis. We found that in mouse cortex and dentate gyrus (DG), BDNF is mainly expressed by nonglial cells (S7 Fig, S8 Fig), which is supported by previous studies [46,47], and this pattern is not altered in AD brains (S7 Fig, S8 Fig).

NTRK2 is expressed in the cortex and DG, again mainly in nonglial cells but few GFAP-positive astrocytes were NTRK2-positive in both the wild-type and AD mouse brains with no clear change in the expression pattern between healthy and diseased brains (S7 Fig). Overall, NTRK2 is expressed mainly by neurons but also few astrocytes and microglia (S8 Fig). We found that p75/NTR is mainly expressed in neurons in the cortex and the DG, but NSC niche in the DG (subgranular zone[(SGZ)]) does not express p75/NTR (S7 Fig, S9 Fig). In both wild-type and APP/PS1dE9 mouse, we could detect BDNF, NTRK2, and p75/NTR expression; however, increased number of GFAP-positive astrocytes AD brains did not correlate with the expression of these proteins, suggesting that BDNF signaling might not regulate NSC proliferation in mouse brains.

To test this hypothesis, we investigated the effect of BDNF in wild-type and AD mouse brains by injecting BDNF into the mouse brains (S7 Fig, S10 Fig). BDNF injection was performed in one hemisphere of the mouse brain, whereas the other hemisphere was used as a control with PBS injection. We found that BDNF injection increased the overall proliferation levels in the brain compared with PBS-injected hemispheres in both wild-type and APP/PS1dE9 brains (S7 Fig). In addition, we also checked the cell types that increase their proliferation levels.

To identify the BDNF-responsive proliferating cells, we performed co-staining with astrocyte marker GFAP and found that the cells positive for Ki67 (antigen identified by monoclonal antibody Ki-67, a marker for proliferating cells) are GFAP negative, suggesting that BDNF-responsive proliferating cells are not astrocytes (S7 Fig, S10 Fig). Next, we performed co-staining of Ki67 with microglial marker Iba1 (ionized calcium binding adaptor molecule 1) and found that almost all Ki67-positive cells are Iba1-positive, suggesting that BDNF induces microglial proliferation resulting in microgliosis but not NSC proliferation (S7 Fig, S10 Fig). This finding is consistent with previous reports [7,48,49]. Additionally, because of the lack of p75/NTR expression in NSCs present in the SGZ of the mouse hippocampus, BDNF/p75NTR signaling, which enhances proliferative output of NSCs and neurogenesis in zebrafish brain, is not an active signaling mechanism in mouse brains. With these results, we propose that zebrafish utilizes special circuit mechanism that uses serotonin-BDNF signaling to enhance the NSC plasticity and to induce neurogenesis through neuronal intermediates; however, BDNF signaling is not regulating NSC plasticity and neurogenesis in mammalian brains whereas its effect is mainly on the neuronal survival and regulation of immune reaction [36,41,49–52].

## Discussion

Our results identify a previously uncharacterized regulatory circuit mechanism that involves neuronal intermediates for NSC plasticity of zebrafish brain in AD conditions. This mechanism involves the suppressive effect of serotonin on periventricular neurons that express *bdnf*, which directly regulates a subset of NSCs expressing BDNF receptor *ngfra*. amyloid-induced IL4 suppresses the initial serotonergic output and therefore potentiates the *bdnf* expression and stem-cell proliferation. We propose that induced expression of IL4 generates a disease-associated NSC niche environment that is supportive of NSC proliferation.

Serotonin affects neurogenesis, stem-cell proliferation, and normal functioning of neural circuitry; however, its effects on NSCs and neurogenesis are controversial [53]. In zebrafish, serotonin positively affects NSC proliferation in midbrain but not the hypothalamus [54] and promotes spinal motor neuron regeneration [55]. Although depletion of serotonin reduces adult neurogenic outcome in rats [56], elevated levels of serotonin in serotonin transporter (SERT)-deficient mice did not affect NSC proliferation and neurogenesis [57,58]. In mesencephalic and hippocampal progenitors of mouse brain, inhibition of serotonin receptors or knocking out serotonin-producing enzymes to generate hyposerotonergic phenotypes had positive effects on neurogenesis [59–62] ascertaining a negative role for serotonin on NSC plasticity. These findings indicate that the effects of serotonin on NSCs are context dependent and may run through intermediary secondary signaling mechanisms, which supports our findings. Additionally, in the majority of the studies, the specific receptors for serotonin were not investigated in NSCs; therefore, our study provides a detailed delineation of the signaling cascades that link serotonergic input to the regulation of stem cell proliferation via intermediates in our study by BDNF.

The interaction between serotonin and BDNF is partially understood. In stress-related disorders, depletion of serotonin reduces brain BDNF levels [63–65]. On the contrary, hyposerotonergic mouse (*Tph2*−/− or *Pet1*−/−) showed increased BDNF levels in the hippocampus and prefrontal cortex [60–62,66], and humans with SERT deficiency (hyperserotonin) reduced the availability of BDNF [67], which enhances NSCs proliferation and neurogenesis [68,69]. Our findings support the negative role of serotonin on BDNF signaling and NSC plasticity.

The effects of BDNF in AD are mainly on neuronal survival rather than neurogenesis or NSC plasticity. Although BDNF expression correlates with increased neuronal survival [70–72] and may have positive impact on increased net hippocampal neurogenesis and better cognitive functioning [73,74], several studies showed that these effects are not directly related to enhanced neurogenesis [48,49]. In fact, in a recent study, in 5X-FAD AD model of mice, increasing adult neurogenesis and simultaneous BDNF treatment increases cognitive output yet adult neurogenesis or BDNF alone are not sufficient to do so [7], suggesting that a BDNF-dependent neuronal survival cascade is required to counteract the symptoms of AD after an independent stimulation of neurogenesis. In zebrafish, in contrast to mammals, BDNF directly regulates NSC plasticity and neurogenesis. Our results in APP/PS1dE9 mice also supported the findings in mice [7] and indicate that zebrafish has an intrinsic and natural ability to kick-start NSC proliferation and neurogenesis, which could teach us how to modulate mammalian brains to initiate “regeneration” of lost neurons in AD.

BDNF expression is abundant in mammalian brains [75], and its main receptor is *TrkB/Ntrk2*, which is predominantly expressed in neurons [76] in the cortex and DG as well as in few astrocytes and microglia as determined by single-cell sequencing [46,47]. BDNF also has binding affinity towards p75 receptor, which is primarily expressed by neurons in mammals [46,47]. Yet, in zebrafish, *ngfra* was predominantly expressed in NSCs and were responsible for NSC plasticity. Zebrafish glial cells responded to BDNF via *ngfra* receptor and activated proliferation and neurogenesis; whereas, mouse glial cells responded to BDNF by reactive astrogliosis and microgliosis. Therefore, our results also propose that the BDNF activity could be specialized to distinct receptor signaling pathways in vertebrate brains. For instance, BDNF-NGFRA signaling could be a factor underlying the regenerative and plastic nature of the NSCs in regenerating organisms such as zebrafish but not in nonregenerating organisms like mammals, in which BDNF mostly acts through TrkB/Ntrk2 signaling. Further studies of this regulatory mechanism in other regenerating organisms would be instrumental in testing our hypothesis. Furthermore, inducing expression of *ngfra* in stem-cell populations in mammalian brains could be a potential way to impose neurogenic plasticity to astrocytes in AD conditions.

We found that IL4 suppresses the serotonin production in neurons that do not express *il4r*, suggesting that IL4 regulates serotonin availability indirectly. Indeed, proinflammatory cytokines such as IL-1β, TNFα, IFNα, and IFNγ can increase 5-HT levels and the expression of 5-HT transporter SERT, which positively correlates with 5-HT levels in the brain [77–83]. Theoretically, anti-inflammatory factors would be expected to reduce 5-HT levels in the brain because of their role in suppression of proinflammatory factor expression. Indeed, IL4 reduces the 5-HT or SERT levels [84–87], supporting our findings. Therefore, in the adult zebrafish brain, AD conditions elicit an inflammatory regulation by IL4 on serotonin, which translates into a neurotransmitter response in a specific neuronal subtype that directly regulates a subpopulation of NSCs.

We identified 2 functionally distinct populations of NSCs that are both responsive to IL4 but through different receptor signaling (IL4R/STAT6 and BDNF/NGFRA) and through distinct gene regulation (Fig 5D and 5E). This finding suggests that NSC heterogeneity is an important determinant of how a certain type of neuropathology and a particular signaling pathway would affect certain subtypes of cells differentially (e.g., in AD conditions, the regulation of proliferation by IL4 in 2 distinct modes [Fig 6]). This understanding would be instrumental in designing targeted therapy options for AD in humans and may lead to more detailed analyses of NSC subtypes in experimental mammalian models of AD. Additionally, the role of IL4 and serotonin in regulating diverse signaling mechanisms in NSCs opens up interesting research avenues as to whether modulation of neuroinflammatory milieu in mammalian brains would kick-start a “regeneration” response by mobilizing the endogenous reservoir of NSCs, which is a controversial subject [8,12–14,23,88,89]. We propose that zebrafish can be used to address neurogenesis-related questions in disease conditions and could serve as a useful experimental model for investigating the molecular mechanisms of NSC plasticity.

## Materials and methods

### Ethics statement

All animal experiments were carried out in accordance with the animal experimentation permits of Referate 25/26 (Veterinärwesen, Pharmazie, und GMP) of the state administration office of Saxony, Germany (Landesdirektion Sachsen) and the ethical commission of TU Dresden (Kommission für Tierversuche). Zebrafish were maintained and handled according to the guidelines [90–93] and EU Directive 2010/63 Article 33 and Annex III (permit numbers: TVV-35/2016 and TVV-52/2015). For mouse studies, APP/PS1 mice was kept and bred according to the established protocols (permit number: TVV 87/2016).

### Animal handling and husbandry

For zebrafish studies, 6- to 8-month-old wild-type AB strain, Tg(her4.1:GFP) and Tg(NFkB:GFP) fish of both genders were used. For each set of experiments, the same clutches of fish were randomly distributed for different experimental groups. For mouse studies, 12-month-old transgenic mice and wild-type littermates (males only) were used for the experiments.

### Peptide synthesis, cerebroventricular microinjection in adult zebrafish

Aβ42 synthesis was performed using the standard 9-fluorenylmethoxycarbonyl (Fmoc) (Merck, Darmstadt, Germany) chemistry with 2-(1H-benzotriazol-1-yl)-1,1,3,3-tetramethyluronoiumhexafluorphosphate (HBTU) (Merck, Darmstadt, Germany) as the coupling reagent on an automated solid-phase peptide synthesizer as previously described in detail [29,30,32]. Cerebroventricular microinjection (CVMI) in adult zebrafish brain were performed as previously described [29, 30, 32]. In short, zebrafish were first anesthetized until the opercular movement was ceased. A small incision was created using the tip of a 30 G needle in the skull above the optic tectum without damaging the brain tissue. A glass capillary (WPI, Saratosa, FL) loaded with the injection solution was inserted through the incision site such that the tip of the glass capillary is oriented toward the telencephalon. Injection was performed with optimum microinjector parameters as previously described. PBS (1μl), Aβ42 (1 μl, 20 μM), IL4 (1 μl, 10 μg/ml, ThermoFisher Scientific, Darmstadt, Germany), BDNF (1 μl, 100ng/ml, R&D Systems, Minneapolis, MN), 5-HT (1 μl, 100 μM, Merck, Darmstadt, Germany), and *ngfra* morpholino (1μl, 10uM) were injected. See S1 Table for further information.

### BrdU treatment

Experimental zebrafish were immersed in freshly prepared 10 mM BrdU (Sigma-Aldrich, Darmstadt, Germany) solution in E3 for 8 hours per day at 2 and 3 dpi. For all BrdU-chase experiments, fish were sacrificed at 14 dpi, and brain samples were subjected to histological preparations.

### Zebrafish tissue preparation, IHC and ISH

Zebrafish were sacrificed at appropriate time points, the heads were dissected, and subjected for overnight fixation at 4°C using 2% paraformaldehyde (Merck, Darmstadt, Germany). For cryoprotection and decalcification, the heads were incubated in 20% Sucrose (Merck, Darmstadt, Germany) / 20% ethylenediaminetetraacetic acid (EDTA, Merck, Darmstadt, Germany) solution overnight at 4°C. The following day, heads were embedded in cryoprotectant sectioning resin in 20% Sucrose (Merck, Darmstadt, Germany) / 7.5% Gelatin (Merck, Darmstadt, Germany) and stored at −80°C. These samples were cryosectioned into 12-μm thick sections using a cryostat and collected onto glass slides which were then stored at −20°C.

For IHC, the sections were dried at room temperature, followed by washing steps in PBS with 0.03% Triton X-100 (Merck, Darmstadt, Germany) (PBSTx). The slides were then incubated overnight in desired primary antibodies at 4°C. On the next day, the slides were washed 3 times with PBSTx and incubated for 2 hours in appropriate secondary antibodies at room temperature. The slides were then washed several times before mounting using aquamount (Thermo Fisher Scientific, Darmstadt, Germany). For TUNEL assay to detect apoptotic cells, ApopTag Red In Situ apoptosis detection kit (Merck, Darmstadt, Germany) was used.

For ISH, digoxigenin (DIG, Roche, Basel, Switzerland)-labeled probes were generated using the DIG RNA labeling kit (Roche, Basel, Switzerland) from the clones constructed with transcripts from the following genes: *htr1aa*, *htr1ab*, *htr1d*, *bdnf*, *ntrk2a*, *ngfra*. The detailed protocol for zebrafish tissue preparations, cryosectioning, IHC, and ISH has been previously described [30,94,95]. See S1 Table for further information on reagents.

### Quantitative real-time PCR

Total RNA was isolated from the telencephalon of adult zebrafish from each experimental group after 1 dpi. A total of 1 μg RNA from each group was reverse transcribed using the Superscript IV VILO Master mix kit (Invitrogen, Carlsbad, CA) according to the manufacturer’s instructions. The real-time PCR was performed in CFX96 Real-Time System (BIORAD, Hercules, CA) with 10 μl reaction volume comprising of SYBR Green I Master mix (Roche, Basel, Switzerland), 1 uM primers, and cDNA. The qPCR data analysis was performed by relative gene expression method using *beta-actin* as a housekeeping gene. The list of primers used for qPCR is provided in S1 Table.

### BDNF injection into adult mouse brain

For the mouse experiment, 100 ng of BDNF (1 μl, 100μg/ml, R&D Systems, Minneapolis, MN) was injected into one hemisphere of the adult mouse brain, and the other hemisphere was injected with 1 μl PBS as a control. The injection procedure was carried out according to the previously established protocol [96]. The mouse was placed in an induction chamber and anesthetized using a mix of oxygen and isoflurane (Sigma-Aldrich, Darmstadt, Germany) flow. After the animal was recumbent, the gas flow directed to the nosecone of the stereotaxic frame. The mouse was then rapidly positioned on the nosecone to have constant supply of isoflurane and oxygen. The ear bars were fixed to immobilize the head. An analgesic was subcutaneously injected prior to surgery to minimize any possible pain after the recovery from anesthesia. During the entire surgery, the animal was placed on a prewarmed heat pad to prevent hypothermia. To prevent dehydration of cornea and further blinding of mouse, eyes were covered with a protective ointment. All tools used during the surgery were sterilized using ethanol and/or Microzide (Sigma-Aldrich, Darmstadt, Germany). Using bregma as a reference point, the coordinates for the injections into the DG were identified on both sides of the brain, and holes were carefully drilled in the skull. The capillary was then slowly inserted until 1,900 μm of depth from skull level and 1 μl of BDNF (100μg/ml, R&D Systems, Minneapolis, MN) was injected at 200 nl/min. After injection, the capillary was kept inside for 5 minutes to prevent backsplash and then slowly retracted. The same procedure was performed on the other hemisphere to inject 1 μl of PBS. The ear bars were then released, the skin was wetted with PBS to regain the elasticity and stitched. The wound was then treated with a disinfecting solution (Povidone, Sigma, Darmstadt, Germany). Mouse was placed in an individual cage under a red light with water and presoaked food (to make it easier for animal to bite after the surgery). Another injection of analgesic was performed 24 hours postsurgery. Mice were sacrificed at 48 hours after injection and subjected for brain collection and further processing.

### Mouse tissue preparations and stainings

For brain collection, the adult mice were anesthetized with an intraperitoneal injection of a mixture of Ketamine (Bio-Techne GmbH, Wiesbaden, Germany) and Xylazine (Sigma-Aldrich, Darmstadt, Germany) (0.25 mL per 25 g of body weight) and then intracardiacally perfused with 0.9% NaCl (Merck, Darmstadt, Germany) followed by cold freshly prepared 4% PFA (in phosphate buffer [pH 7.4]). After, perfusion brains were harvested and fixed with 4% PFA for 24 hours at 4°C and then washed 3 times with PBS. For cryopreservation of tissue, fixed brains were further incubated overnight in 30% Sucrose (Merck, Darmstadt, Germany) solution in PBS at 4°C. Free-floating sections of 40 μm thickness were prepared using microtome (Leica SM2010R, Nussloch, Germany) and were collected in 6 consecutive series in a cryopreservation solution in a 24-well plate. Sections were then stored at −20°C for further experiments. Prior to IHC, the free-floating sections were washed in PBS 3 times, blocked in 10% Donkey or Goat Serum (Abcam, Cambridge, UK) with 0.2% Triton X-100 (Merck, Darmstadt, Germany) for 1 hour at room temperature, and incubated overnight at 4°C with the desired primary antibody of defined dilution in 3% Donkey or Goat Serum (Abcam, Cambridge, UK) with 0.2% Triton X-100. Sections were washed 3 times within 1 hour and incubated for another 4 hours at room temperature with the correct secondary antibody coupled to a desired fluorophore. After a short wash, samples were then incubated in DAPI (Invitrogen, Carlsbad, CA) diluted in PBS (1:5,000) for 10 minutes. Another 3 washing steps were done, and samples were then mounted on the charged glass slides (SuperFrostTM, Merck, Darmstadt, Germany). After mounting, slides were left to dry and covered with a coverslip using Aqua Mount. See S1 Table for further information on reagents.

### Imaging and quantifications

Images were acquired using ZEN software (Carl Zeiss, Jena, Germany) on a Zeiss fluorescent microscope with ApoTome or Zeiss AxioImager Z1 (Oberkochen, Germany) and analyzed using ZEN (Carl Zeiss, Jena, Germany) or ImageJ (https://imagej.nih.gov/ij/) software. Quantifications were performed in double blind fashion. Stereological quantifications in zebrafish were performed by manual counting on one-third of the whole sections from the telencephalon (caudal olfactory bulb until the rostral optic tectum). Quantifications for serotonergic densities were performed using a 3D object counter module of ImageJ (https://imagej.nih.gov/ij/) software. At least 4 animals with a minimum 5 to 12 histological sections per animal were used for stereological analyses in all ISH and IHC stainings unless otherwise stated.

### Statistical analyses

All experiments were performed at least with 2 replicates. For comparison of 2 particular experimental groups, a two-tailed Student t test was performed. Statistical analyses were performed using Microsoft Excel (Microsoft Corporation, Redmond, WA). *p*-values less than 0.05 were considered significant. Error bars in the figures indicate the standard deviation. Significance indicated by * (*p <* 0.05), ** (*p <* 0.01), and *** (*p <* 0.001), n.s. (not significant, *p >* 0.05). No sample set was excluded from the analyses unless the histological sections were damaged severely during the acquisition of the sections (constitutes less than 4% of all sections analyzed).

### Cell dissociation, sorting, whole transcriptome and single-cell sequencing

The telencephalon was dissected using ice-cold PBS, and cell dissociation was performed using the Neural Tissue Dissociation Kit (Miltenyi Biotec, Bergisch Gladbach, Germany) following the manufacturer’s instructions and as described previously [30]. Deep sequencing for whole transcriptome was performed as described [20,30]. For single-cell sequencing, isolated cells were passed through a 40-μm cell strainer. Viability indicator (propidium iodide, Merck, Darmstadt, Germany) and GFP were used to sort live cells. Cell suspension was loaded into the 10X Chromium system (10X Genomics, Pleasanton, CA) [97]. The 10X libraries were prepared as per the manufacturer’s instructions. The raw sequencing data were processed by the cell ranger software provided by the 10X genomics with the default options. The reads were aligned to zebrafish reference transcriptome (ENSEMBL Zv10, release 91). Analyses matrices were used as input for downstream data analysis by Seurat (https://satijalab.org/seurat/) [98]. Analyses were performed as described [34,99].

### Data analysis by Seurat

Read10X function was used for generated matrices. Cells that contain more than 1,000 to 15,000 UMI and 500 to 2,500 unique genes were taken into consideration. Multiplet cells and cells with more than 6% mitochondrial genes were removed from further analyses. Genes found in less than 5 cells were removed from analyses as well. The data were normalized by using “LogNormalize” method and were scaled with “scale.factor = 1e4”. Variable genes were detected with FindVariableGenes. The top 1,000 most variable genes from each sample were merged, and canonical correlation analysis (CCA) was performed on the variable genes. A merged Seurat object was created with RunMultiCCA function, using num.ccs = 30. The canonical correlation strength was calculated using num.dims = 1:30. The reduction was performed by reduction.type = “cca”, dims.use = 1:30. The samples were aligned using dims.align = 1:30. The cell clusters were found using aligned CCA and 1:30 dims, with resolution 1.0. Clusters were shown on 2D using tSNE function. We identified the main cell clusters and subclusters of neurons and PCs as previously described [34].

### Cell type identification by machine learning, pathway analyses, and interaction mapping

We used all marker genes with False Detection Rate (FDR) < 0.1 for Gene Ontology (GO) analysis and KEGG-pathway analysis using GOStats (1.7.4) [100] and GSEABase (1.40.1), *p* < 0.05 as thresholds. To determine the DEGs, we used FindMarkers function using cell cluster that have at least 3 cells from all samples. Then, we used *p* <0.05 for significantly expressed genes.

Cell types in serotonin single-cell sequencing data set were identified by using machine learning algorithm. Top-5 marker genes for each cells for 3 samples (PBS, AB42, and IL4 as previously described [34]) were determined. These genes were used to train RandomForest algorithm (https://cran.r-project.org/web/packages/randomForest/index.html) to learn cell types from existing samples. The cells in 5-HT treated samples were predicted accordingly. We identified marker genes, DEGs, and interaction maps as described before [34]. We also performed Seurat (https://satijalab.org/seurat/) analyses and determined that the experimental conclusions are consistent regardless of the method used (S5 Fig).

To identify DEGs in specific cell types, we added new “idents” to the R-object as the following: Treatment (PBS, AB42, IL4, 5HT, or AB42/IL4 cells together)_Main Cell Types, (Im, PC, NN, OPC/OD1 or OPC/OD_2)_receptor name. The DEGs in each of these cells were identified after each treatment using FindMarkers function in Seurat. *p* < 0.05 and |avg_logFC| ≥ 0.25 were used for significantly expressed genes; *p* < 0.05 was used for overrepresented GO terms and KEGG pathways as described [34]. Selection from DEGs for *htr1d*-positive cells were performed with the absolute log-fold change criterion of >0.25.

### Electrophysiology

Adult zebrafish were cold-anesthetized in a slush of a frozen extracellular solution. The skull was removed to allow access to the brain. The brain was dissected out carefully and transferred to a recording chamber that was continuously perfused with an extracellular solution containing 135.2 mM NaCl (Merck, Darmstadt, Germany), 2.9 mM KCl (Merck, Darmstadt, Germany), 2.1 mM CaCl_2_ (Merck, Darmstadt, Germany), 10 mM HEPES (Merck, Darmstadt, Germany), and 10 mM glucose (pH 7.8; 290 mOsm, (Merck, Darmstadt, Germany). For whole-cell intracellular recordings of her4.1:GFP cells in voltage-clamp mode, electrodes (resistance, 3–5 MΩ) were pulled from borosilicate glass (outer diameter, 1.5 mm; inner diameter, 0.87 mm; Hilgenberg GmbH, Malsfeld, Germany) on a micropipette puller (Sutter Instruments, Novato, CA) and filled with an intracellular solution containing 120 mM K-gluconate (Merck, Darmstadt, Germany), 5 mM KCl (Merck, Darmstadt, Germany), 10 mM HEPES (Merck, Darmstadt, Germany), 4 mM Mg_2_ATP (Merck, Darmstadt, Germany), 0.3 mM Na_4_GTP, and 10 mM Na-phosphocreatine (pH 7.4; 275 mOsm, Merck, Darmstadt, Germany). GFP-positive cells were visualized using a microscope (LNscope; Luigs & Neumann, Ratingen, Germany) equipped with a CCD camera (Lumenera) and were then targeted specifically. Intracellular patch-clamp electrodes were advanced to cells using a motorized micromanipulator (Luigs & Neumann, Ratingen, Germany) while applying constant positive pressure. Intracellular signals were amplified with a MultiClamp 700B intracellular amplifier (Molecular Devices, San Jose, CA). All cells were clamped at –70 mV throughout all recordings. All experiments were performed at room temperature (23°C ± 1°C). Serotonin (100 μM; Sigma-Aldrich, Darmstadt, Germany) was added to the physiological solution in all recordings, the recorded events were detected and analyzed using AxoGraph (version X 1.5.4; AxoGraph Scientific, Sydney, Australia) or Clampfit (version 10.6; Molecular Devices, San Jose, CA).

## Supporting information

S1 Fig Analyses of whole-transcriptome sequencing after IL4 treatment. (A) Sample correlation heat map and principle component analyses. (B, C) KEGG enriched pathways as list (B) and pie chart (C). (D) GO terms for biological process. (E) GO terms for molecular function. (F) Tryptophan metabolism in detail. Green: down-regulated enzymes, red: up-regulated enzymes after IL4. Related to Fig 1. See S1 Data and S2 Data for supporting information. GO, gene ontology; IL4, interleukin-4; KEGG, Kyoto Encyclopedia of Genes and Genomes.
(JPG)

S2 Fig Amyloid or IL4 do not cause death of 5-HT neurons. (A–C) 5-HT immunostaining in caudal regions of the adult zebrafish brain: Superior Raphe (A, Aʹ), pineal stalk (B), and paraventricular organ (PVO) of hypothalamus (C). (D–I) 5-HT and TUNEL stainings in control (D, E), Aβ42-injected (F, G), and IL4-injected (H, I) zebrafish brains. (D,F,H) PVO region; (E, G, I) superior raphe. (F1, F2) Higher magnification images of the boxes in panel F. (G1) Higher magnification of the box in panel G. Scale bars equal 50 μM. Related to Fig 1. Aβ42, amyloid-beta42; IL4, interleukin-4; PVO, paraventricular organ; TUNEL, terminal deoxynucleotidyl transferase dUTP nick end labeling; 5-HT, serotonin.
(JPG)

S3 Fig Aβ42 and IL4 antagonize the indirect effect of 5-HT on neural stem cell plasticity. (A–D) IHC for S100β and PCNA on control (A), 5-HT-injected (B), 5-HT + Aβ42-injected (C), and 5-HT + IL4-injected (D) zebrafish brains. (E) Quantification of proliferating glial cells in all conditions. (F) Read numbers of all serotonin receptors in her4.1+ cellspositive cells (PCs) in the adult zebrafish telencephalon as a graphical representation that is derived from deep sequencing results. Glial markers *gfap* and *s100* are given as positive controls. (G) ISH panels of *htr1aa*, *htr1ab*, and *htr1d*. Note that progenitor cells/glia do not express serotonin receptors. (H) Electrophysiology in 1-month-old her4.1:GFP zebrafish telencephalon. GFP+ cellspositive cells (NSCs) were patched with electrodes, and the effect of 5-HT on membrane polarization was measured. NSCs are not responsive to 5-HT but periventricular neurons that are GFP-negative are. *n* > 9 for electrophysiology experiments. Scale bars equal 100 μM. Related to Fig 2. See S7 Data for supporting information. Aβ42, amyloid-beta42; IHC, immunohistochemistry; IL4, interleukin-4; NSC, neural stem cell; PC, progenitor cell; PCNA, proliferation cell nuclear antigen; S100β,; 5-HT, serotonin.
(JPG)

S4 Fig Single-cell sequencing analyses of adult zebrafish telencephalon after serotonin treatment. (A) Schematic workflow for single-cell sequencing. (B) Quality control indicators of single-cell sequencing data: VLN plots for principal component analyses, variable gene plots, distribution plots for number of genes (nGene), number of reads (nUMI), % of mitochondrial genes (%mito), and gene plots for %mito, nGene, and %GFP (from sorted her4.1-GFP cells). (C) Primary tSNE feature plots indicating major cell clusters with canonical markers: *sypb* and *gap43* for neurons, *olig2* and *aplnrb* for oligodendrocytes, *gfap* and her4 for glia, *pfn1* and *lcp1* for immune cells. (D) Primary heat map for top 40 marker genes of neurons, glia, oligodendrocytes, and immune cells. (E) Classification of major cell clusters for their identities based on markers. (F) Feature plots for *htr1* and *il4r* expression. Note that *htr1*-positive cells are neurons whereas *il4r*-positive cells are glia. (G) Strategy for isolating *htr1*-expressing cells from tSNE plot and subsequent differential expression analyses. (H) VLN plots for *bdnf*, *ngfra*, and *ntrk2* in major cell types and expression level ratios as pie charts. Related to Fig 3. See S3 Data for supporting information. GFP, green fluorescent protein; tSNE, t-Distributed stochastic neighbor embedding; VLN, violin plot.
(JPG)

S5 Fig Comparison of de novo clustering with Seurat and machine learning paradigm. Cells are color-coded in samples (A), cell clusters predicted by RandomForest (B), and cell clusters identified by Seurat (C) after using all 4 experimental groups together. To use the same neuronal and progenitor clusters we identified before ([34]), we used RandomForest and machine learning (B) in our analyses. By using Seurat (C), cell clusters can also be inferred de novo. The cell clusters and their top marker genes are identical, whereas some cell clusters (e.g., neurons) can be further subdivided depending on the algorithm used. The color codes used in the middle panel are the same colors used in [34]. The colors of PCs are also used in Seurat analyses (A). A few cells from Aβ42 and 5-HT groups do not exist in other groups (control and IL4). These cells express olfactory bulb markers and are contaminations of cells in sample preparation. They cluster separately from all groups we analyzed and are not affecting the biological outcomes of the analyses. Related to Fig 3. See S3 Data for supporting information. Aβ42, amyloid-beta42; IL4, interleukin-4; PC, progenitor cell; 5-HT, serotonin.
(JPG)

S6 Fig Serotonin suppresses and BDNF enhances NFkB signaling in NSCs in zebrafish. (A) In silico interaction map for NTRK2 in Aβ42 versus control, IL4 versus control, and 5-HT versus control comparisons. Black arrows: interactions unchanged with treatment, cyan arrows: interaction lost with treatment, magenta arrows: interaction gained/emerged with the treatment. (B) ISH for *ntrk2* in zebrafish brain. (Bʹ) Close-up image. Note the expression in pvz but not in vz that contains the NSCs. (C) IHC for Ntrk2 protein in zebrafish brain, supporting the ISH results and presence of Ntrk2 in pvz. (D, E) IHC for pAkt in control (D) and BDNF-injected (E) brains. BDNF activates pAkt in pvz but not in vz. (F) ISH for *ngfra* in adult zebrafish telecephalon. (G) IHC for S100β, NfkB-driven GFP, and PCNA in control, Amyloid-injected, IL4-injected, 5-HT-injected, and BDNF-injected brains. Smaller panels under larger images show individual fluorescent channels. (H) Quantification of the relative number of proliferating NSCs that have active NFkB signaling. Scale bars equal 100 μM. Data are represented as mean ± SEM. Related to Fig 4. See S7 Data for supporting information. BDNF, brain-derived neurotrophic factor; GFP, green fluorescent protein; IHC, immunohistochemistry; IL4, interleukin-4; ISH, in situ hybridization; NFkB, nuclear factor 'kappa-light-chain-enhancer' of activated B-cells; NSC, neural stem cell; NTRK2, neurotrophic tyrosine kinase receptor, type 2; pAkt, phosphorylated protein kinase B; PCNA, proliferating cell nuclear antigen; pvz, periventricular zone; S100β, S100 calcium-binding protein B; vz, ventricular zone; 5-HT, serotonin.
(JPG)

S7 Fig BDNF does not induce NSC plasticity in mouse model of AD. (A, B) IHC for GFAP and SOX2 in WT (A) and APP/PS1dE9 mouse brains (B) at 12 months of age. (A1, A2) Single fluorescent channels of panel A. (B1, B2) Single fluorescent channels of panel B. (C–Dʹ) IHC for GFAP and BDNF in WT mouse: (C) cortex, (D) DG. Primed images are higher magnification without DAPI. (E–Fʹ) IHC for GFAP and BDNF in APP/PS1dE9 mouse: (E) cortex, (F) DG. Primed images are higher magnification without DAPI. (G–Hʹ) IHC for GFAP and NTRK2 in WT mouse: (G) cortex, (H) DG. Primed images are higher magnification without DAPI. (I–Jʹ) IHC for GFAP and NTRK2 in APP/PS1dE9 mouse: (I) cortex, (J) DG. Primed images are higher magnification without DAPI. (K–Lʹ) IHC for NeuN and p75/NTR in WT mouse: (K) cortex, (L) DG. Primed images are higher magnification without DAPI. (M–Nʹ) IHC for NeuN and p75/NTR in APP/PS1dE9 mouse: (M) cortex, (N) DG. Primed images are higher magnification without DAPI. (O–R2) IHC for Ki67 and GFAP in WT mouse PBS-injected hemisphere (O), wt mouse BDNF-injected hemisphere (P), APP/PS1dE9 mouse PBS-injected hemisphere (Q), APP/PS1dE9 mouse BDNF-injected hemisphere (R). (S–V2) IHC for Ki67 and Iba1 in WT mouse PBS-injected hemisphere (S), WT mouse BDNF-injected hemisphere (T), APP/PS1dE9 mouse PBS-injected hemisphere (U), APP/PS1dE9 mouse BDNF-injected hemisphere (V). (W) High-magnification image of Ki67 and GFAP staining on DG of BDNF-injected hemisphere of APP/PS1dE9 mouse. GFAP cells do not proliferate after BDNF injection. (X) High-magnification image of Ki67 and Iba1 staining on DG of BDNF-injected hemisphere of APP/PS1dE9 mouse. Most Ki67-positive cells overlap with Iba1 staining after BDNF injection. *n* = 2 animals used for analyses. Scale bars equal 100 μM. Related to Fig 4. AD, Alzheimer disease; BDNF, brain-derived neurotrophic factor; DG, dentate gyrus; GFAP, glial fibrillary acidic protein; Iba1, ionized calcium binding adaptor molecule 1; IHC, immunohistochemistry; Ki67, antigen identified by monoclonal antibody Ki-67; NeuN, Fox-3, Rbfox3, or Hexaribonucleotide binding protein 3; NSC, neural stem cell; NTRK2, neurotrophic tyrosine kinase, receptor, type 2; SOX2, (sex determining region Y)-box transcription factor 2; wt, wild type.
(JPG)

S8 Fig BDNF and NTRK2 expression in mouse brain. (A, Aʹ) IHC for amyloid plaques (4G8) in wild-type mouse hippocampus. (B–F) IHC for GFAP, SOX2, and BDNF in wild-type mouse brains. (G, Gʹ) IHC for amyloid plaques (4G8) in APP/PS1dE9 mouse hippocampus. (H–L) IHC for GFAP, SOX2, and BDNF in APP/PS1dE9 mouse brains. Mice were at the age of 12 months. (M) IHC for GFAP and NTRK2 in wild-type mouse DG. (Mʹ) Single fluorescent channel for NTRK2. (N) High magnification of merged image. (Nʹ) Single fluorescent channel for NTRK2 in panel N. (O) IHC for Iba1 and NTRK2. (Oʹ) Single fluorescent channel for NTRK2 in panel O. (Oʺ) single fluorescent channel for Iba1 in panel O. (P) IHC for NTRK2 and NeuN. Single channel in red is NTRK2. Scale bars equal 100 μM. Related to Figs 4 and 5. BDNF, brain-derived neurotrophic factor; DG, dentate gyrus; GFAP, glial fibrillary acidic protein; Iba1, ionized calcium binding adaptor molecule 1; IHC, immunohistochemistry; NeuN, Fox-3, Rbfox3, or Hexaribonucleotide binding protein 3; IHC, immunohistochemistry;; NTRK2, neurotrophic tyrosine kinase, receptor, type 2; SOX2, (sex determining region Y)-box transcription factor 2.
(JPG)

S9 Fig p75/NTR expression in mouse brain. (A–C) IHC for p75/NTR (NGFR) in wild-type cortex (A), DG (B), and SVZ (C). (D–K) IHC for p75/NTR and NeuN in cortex (D) and DG (H). (E–G) High-magnification image from panel D. (I–K) High-magnification image from panel H. (L–S) IHC for p75/NTR and NeuN in cortex (L) and DG (P) of APP/PS1dE9 mouse. (M–O) High-magnification image from panel L. (Q–S) High-magnification image from panel P. Scale bars equal 100 μM. Related to Figs 4 and 5. DG, dentate gyrus; IHC, immunohistochemistry; NeuN, Fox-3, Rbfox3, or Hexaribonucleotide binding protein 3; NGFR, nerve growth factor receptor; p75/NTR, neurotrophin receptor P75; SVZ, subventricular zone.
(JPG)

S10 Fig BDNF increases the proliferation of microglia but does not affect the astrocytes in the mouse brain. (A, B) IHC for Ki67 and GFAP in wild-type (A) and APP/PS1dE9 (B) mouse brains at 12 months of age after injection of PBS (left hemisphere) and BDNF (right hemisphere). (C) Close-up image of the DG of a BDNF-injected side of the APP/PS1dE9 mouse brain. (D, E) IHC for Ki67 and Iba1 in wild-type (D) and APP/PS1dE9 (E) mouse brains at 12 months of age after injection of PBS (left hemisphere) and BDNF (right hemisphere). (F) Close-up image of the DG of a BDNF-injected side of the APP/PS1dE9 mouse brain. Scale bars equal 100 μM. Related to Figs 4 and 5. BDNF, brain-derived neurotrophic factor; DG, dentate gyrus; Ki67, antigen identified by monoclonal antibody Ki-67; GFAP, glial fibrillary acidic protein; Iba1, ionized calcium binding adaptor molecule 1; IHC, immunohistochemistry; NeuN, Fox-3, Rbfox3, or Hexaribonucleotide binding protein 3; IHC, immunohistochemistry.
(JPG)

S1 Data List of DEGs determined by deep sequencing after injection of IL4 to the adult zebrafish brain. DEG, differentially expressed gene; IL4, interleukin-4.
(XLSX)

S2 Data GO-term and KEGG-pathway analyses for transcriptomics changes in adult zebrafish brain after IL4. GO, gene ontology; IL4, interleukin-4; KEGG, Kyoto Encyclopedia of Genes and Genomes.
(XLSX)

S3 Data Single-cell sequencing quality control data sets: Histograms, VLN plots, tSNE plots, heat maps. tSNE, t-Distributed stochastic neighbor embedding; VLN, violin plot.
(PDF)

S4 Data DEGs in htr1+ cells after single-cell sequencing. DEG, differentially expressed gene.
(XLSX)

S5 Data DEGs in il4r.1+ cells after single-cell sequencing. DEG, differentially expressed gene.
(XLSX)

S6 Data DEGs in ngfra+ cells after single-cell sequencing. DEG, differentially expressed gene.
(XLSX)

S7 Data Excel spreadsheet containing, in separate sheets, the underlying numerical data for panels in Figs 1, 2, 4 and 5 and S3 and S6 Figs. (XLSX)

S1 Table List of materials and reagents used. (XLSX)

## Abbreviations

Aβ42: amyloid-beta42
AD: Alzheimer’s disease
aplnrb: apelin receptor b
APP: amyloid precursor protein
BDNF: brain-derived neurotrophic factor
BrdU: 5-bromo-2'-deoxyuridine
CCA: canonical correlation analysis
cDNA: complementary deoxyribonucleic acid
CVMI: cerebroventricular microinjection
DAPI: 4′,6-diamidino-2-phenylindole
DEG: differentially expressed gene
DG: dentate gyrus
DIG: digoxigenin
dpi: days post injection
eno2: enolase 2
EPSC: excitatory postsynaptic current
fmoc: 9-fluorenylmethoxycarbonyl
gap43: growth associated protein 43
gfap: glial fibrillary acidic protein
GFP: green fluorescent protein
GO: gene ontology
HBTU: 2-(1H-benzotriazol-1-yl)-1,1,3,3-tetramethyluronoiumhexafluorphosphate
her4.1: hairy-related 4, tandem duplicate 1
htr: 5-hydroxytryptamine receptor
HuC/D: ELAV (embryonic lethal, abnormal vision, Drosophila)-like ¾
Iba1: ionized calcium binding adaptor molecule 1
IHC: immunohistochemistry
IL-1β: interleukin-1 beta
IL4: interleukin-4
IFNα: interferon alpha
IFNγ: interferon gamma
ISH: in situ hybridization
KEGG: Kyoto Encyclopedia of Genes and Genomes
Ki67: antigen identified by monoclonal antibody Ki-67
lcp1: lymphocyte cytosolic protein 1 (L-plastin)
map1aa: microtubule-associated protein 1Aa
msi1: musashi RNA-binding protein 1
NeuN: Fox-3, Rbfox3, or Hexaribonucleotide binding protein 3
NFkB: nuclear factor 'kappa-light-chain-enhancer' of activated B-cells
NGFRA: nerve growth factor receptor A
NSC: neural stem cell
ntrk2: neurotrophic tyrosine kinase, receptor, type 2
olig2: oligodendrocyte lineage transcription factor 2
pAkt: phosphorylated protein kinase B
PBS: phosphate-buffered saline
PC: progenitor cell
PCNA: proliferating cell nuclear antigen
pERK: phosphorylated extracellular signal-regulated kinase
pfn1: profilin 1
PVO: periventricular organ
pvz: periventricular zone
p75/NTR: neurotrophin receptor P75
S100β: S100 calcium-binding protein B
SERT: serotonin transporter
SGZ: subgranular zone
pet1: FEV transcription factor, E26 transformation-specific family member
PS1dE9: human presenilin 1 with exon-9-deleted variant
pSTAT6: phosphorylated signal transducer and activator of transcription 6, interleukin-4 induced
PVO: paraventricular organ
SOX2: (sex determining region Y)-box transcription factor 2
sypb: synaptophysin b
TNFα: tumor necrosis factor alpha
tph: tryptophan hydroxylase
tSNE: t-distributed stochastic neighbor embedding
TTX: Tetrodotoxin
TUNEL: terminal deoxynucleotidyl transferase dUTP nick end labeling
UMI: unique molecular identifier
VLN: violin plot
WT: wild type
5-HT: serotonin
5X-FAD: 5 familial AD-linked mutations
