# Supplementary figures and images for: Neuron-glia interaction through Serotonin-BDNF-NGFR axis enables regenerative neurogenesis in Alzheimer’s model of adult zebrafish brain
Source: PLoS Biol. 2020 Jan 6;18(1):e3000585. doi: 10.1371/journal.pbio.3000585 (PMC6964913; doi:10.1371/journal.pbio.3000585)

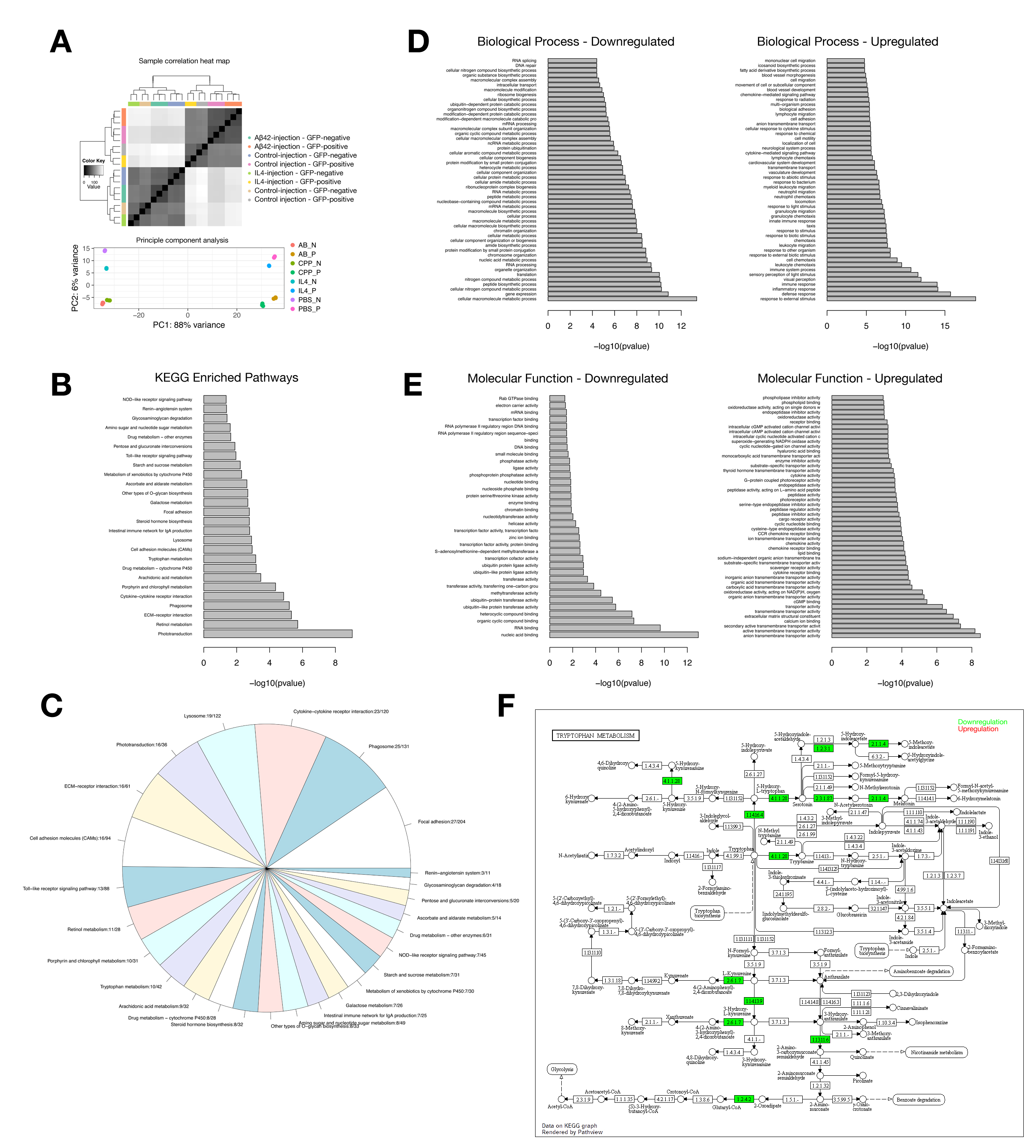

Supplement: S1 Fig — (A) Sample correlation heat map and principle component analyses. (B, C) KEGG enriched pathways as list (B) and pie chart (C). (D) GO terms for biological process. (E) GO terms for molecular function. (F) Tryptophan metabolism in detail. Green: down-regulated enzymes, red: up-regulated enzymes after IL4. Related to Fig 1. See S1 Data and S2 Data for supporting information. GO, gene ontology; IL4, interleukin-4; KEGG, Kyoto Encyclopedia of Genes and Genomes. (JPG) [file pbio.3000585.s001.jpg]

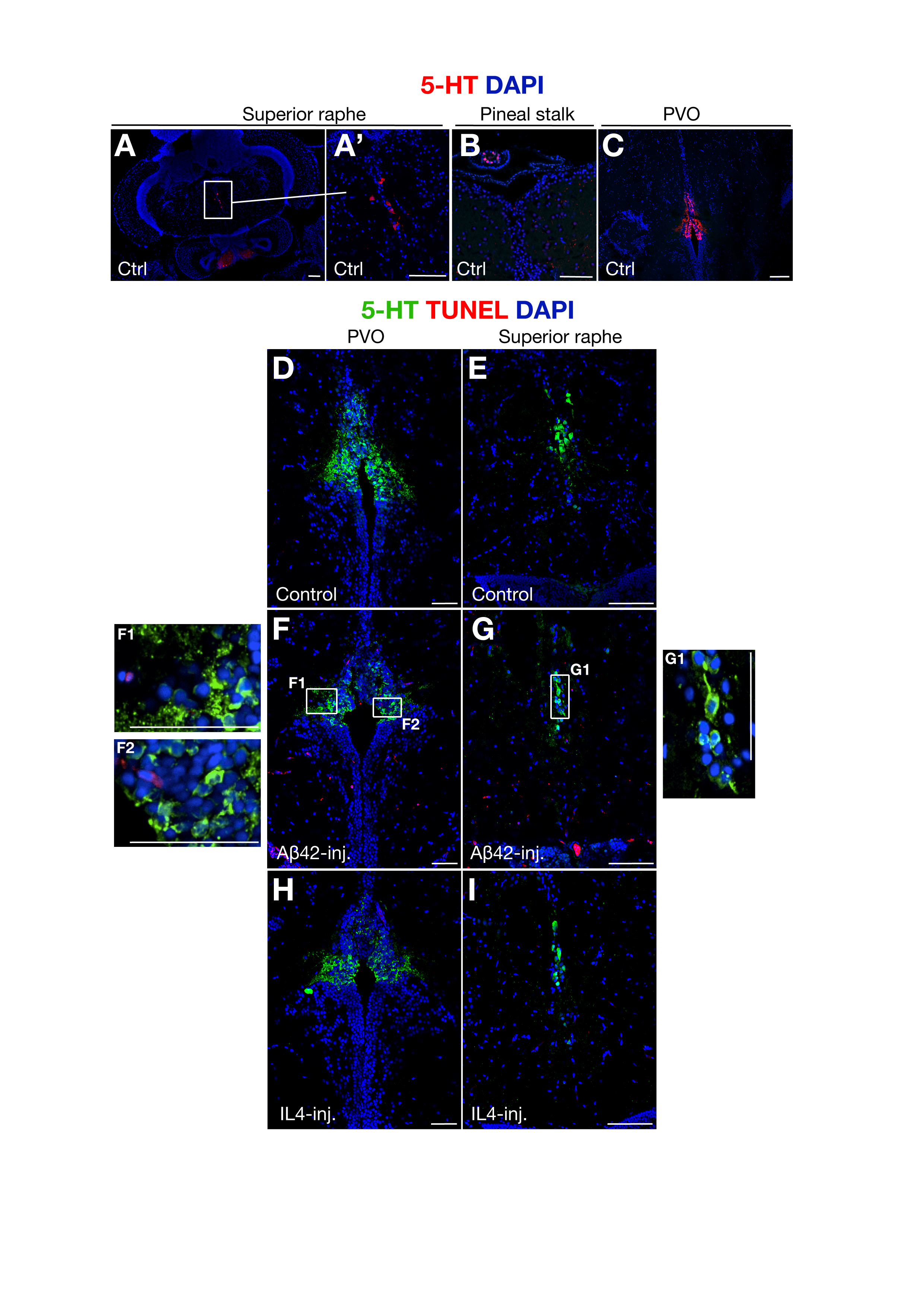

Supplement: S2 Fig — (A–C) 5-HT immunostaining in caudal regions of the adult zebrafish brain: Superior Raphe (A, Aʹ), pineal stalk (B), and paraventricular organ (PVO) of hypothalamus (C). (D–I) 5-HT and TUNEL stainings in control (D, E), Aβ42-injected (F, G), and IL4-injected (H, I) zebrafish brains. (D,F,H) PVO region; (E, G, I) superior raphe. (F1, F2) Higher magnification images of the boxes in panel F. (G1) Higher magnification of the box in panel G. Scale bars equal 50 μM. Related to Fig 1. Aβ42, amyloid-beta42; IL4, interleukin-4; PVO, paraventricular organ; TUNEL, terminal deoxynucleotidyl transferase dUTP nick end labeling; 5-HT, serotonin. (JPG) [file pbio.3000585.s002.jpg]

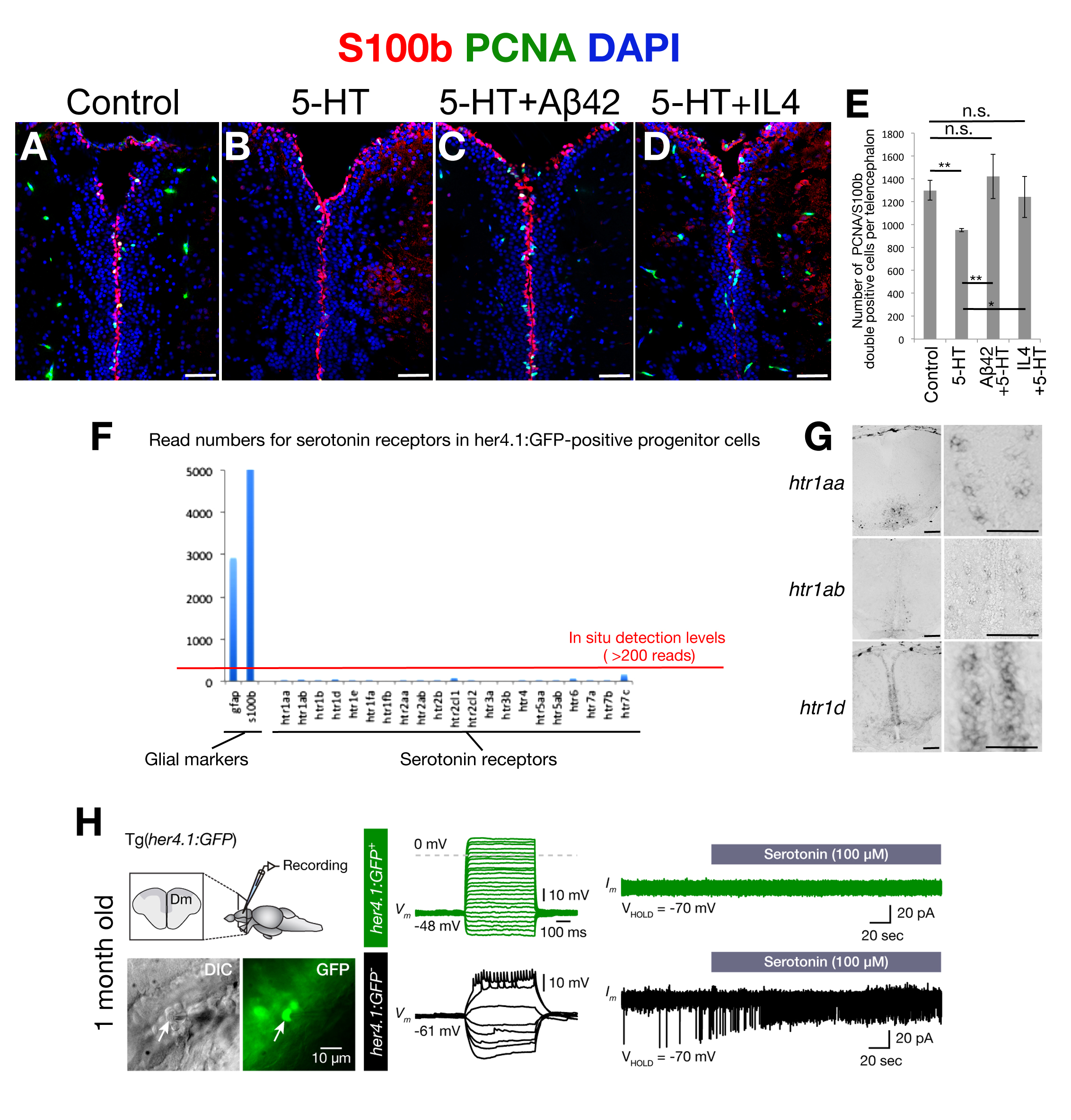

Supplement: S3 Fig — (A–D) IHC for S100β and PCNA on control (A), 5-HT-injected (B), 5-HT + Aβ42-injected (C), and 5-HT + IL4-injected (D) zebrafish brains. (E) Quantification of proliferating glial cells in all conditions. (F) Read numbers of all serotonin receptors in her4.1+ cellspositive cells (PCs) in the adult zebrafish telencephalon as a graphical representation that is derived from deep sequencing results. Glial markers gfap and s100 are given as positive controls. (G) ISH panels of htr1aa, htr1ab, and htr1d. Note that progenitor cells/glia do not express serotonin receptors. (H) Electrophysiology in 1-month-old her4.1:GFP zebrafish telencephalon. GFP+ cellspositive cells (NSCs) were patched with electrodes, and the effect of 5-HT on membrane polarization was measured. NSCs are not responsive to 5-HT but periventricular neurons that are GFP-negative are. n > 9 for electrophysiology experiments. Scale bars equal 100 μM. Related to Fig 2. See S7 Data for supporting information. Aβ42, amyloid-beta42; IHC, immunohistochemistry; IL4, interleukin-4; NSC, neural stem cell; PC, progenitor cell; PCNA, proliferation cell nuclear antigen; S100β,; 5-HT, serotonin. (JPG) [file pbio.3000585.s003.jpg]

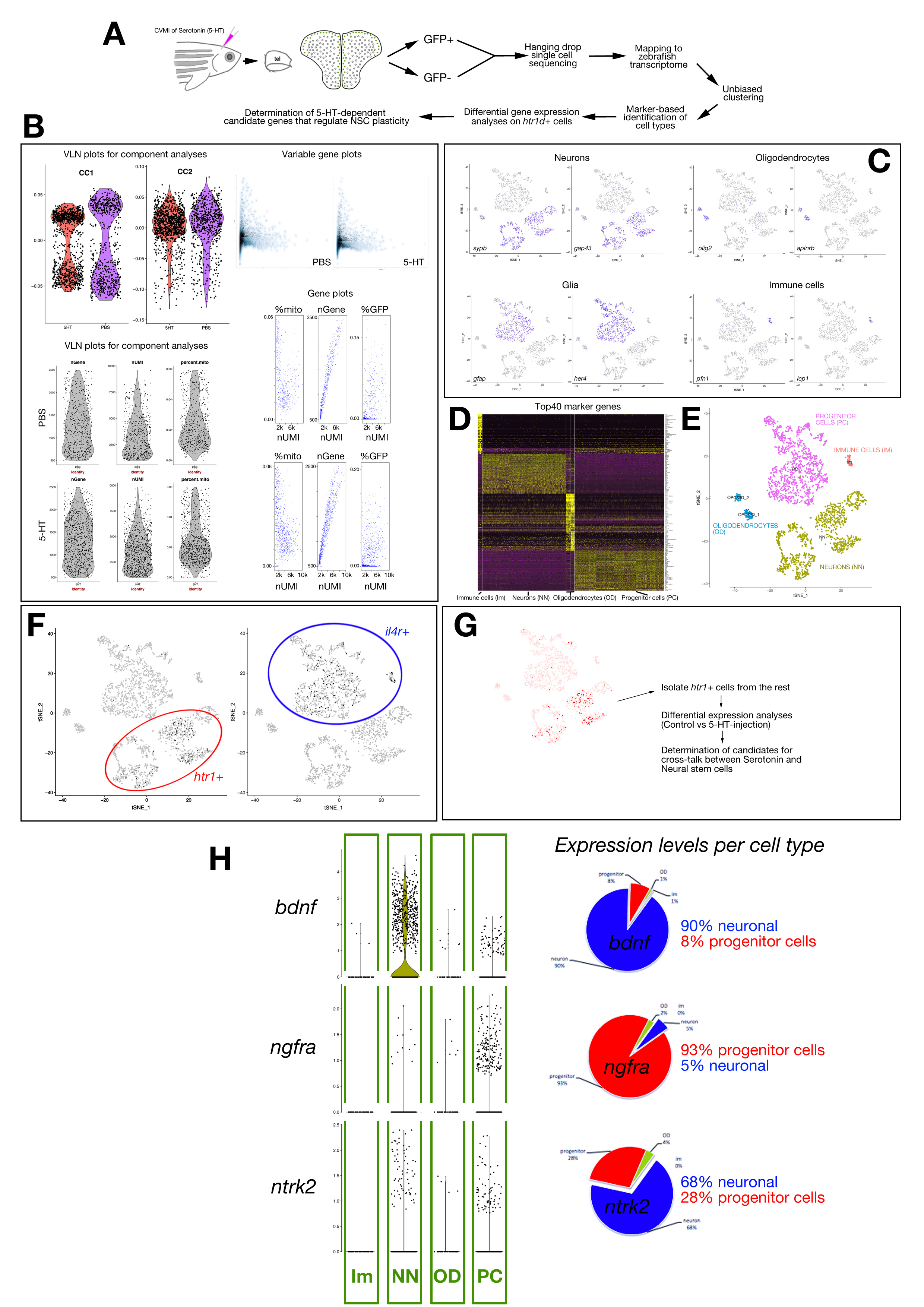

Supplement: S4 Fig — (A) Schematic workflow for single-cell sequencing. (B) Quality control indicators of single-cell sequencing data: VLN plots for principal component analyses, variable gene plots, distribution plots for number of genes (nGene), number of reads (nUMI), % of mitochondrial genes (%mito), and gene plots for %mito, nGene, and %GFP (from sorted her4.1-GFP cells). (C) Primary tSNE feature plots indicating major cell clusters with canonical markers: sypb and gap43 for neurons, olig2 and aplnrb for oligodendrocytes, gfap and her4 for glia, pfn1 and lcp1 for immune cells. (D) Primary heat map for top 40 marker genes of neurons, glia, oligodendrocytes, and immune cells. (E) Classification of major cell clusters for their identities based on markers. (F) Feature plots for htr1 and il4r expression. Note that htr1-positive cells are neurons whereas il4r-positive cells are glia. (G) Strategy for isolating htr1-expressing cells from tSNE plot and subsequent differential expression analyses. (H) VLN plots for bdnf, ngfra, and ntrk2 in major cell types and expression level ratios as pie charts. Related to Fig 3. See S3 Data for supporting information. GFP, green fluorescent protein; tSNE, t-Distributed stochastic neighbor embedding; VLN, violin plot. (JPG) [file pbio.3000585.s004.jpg]

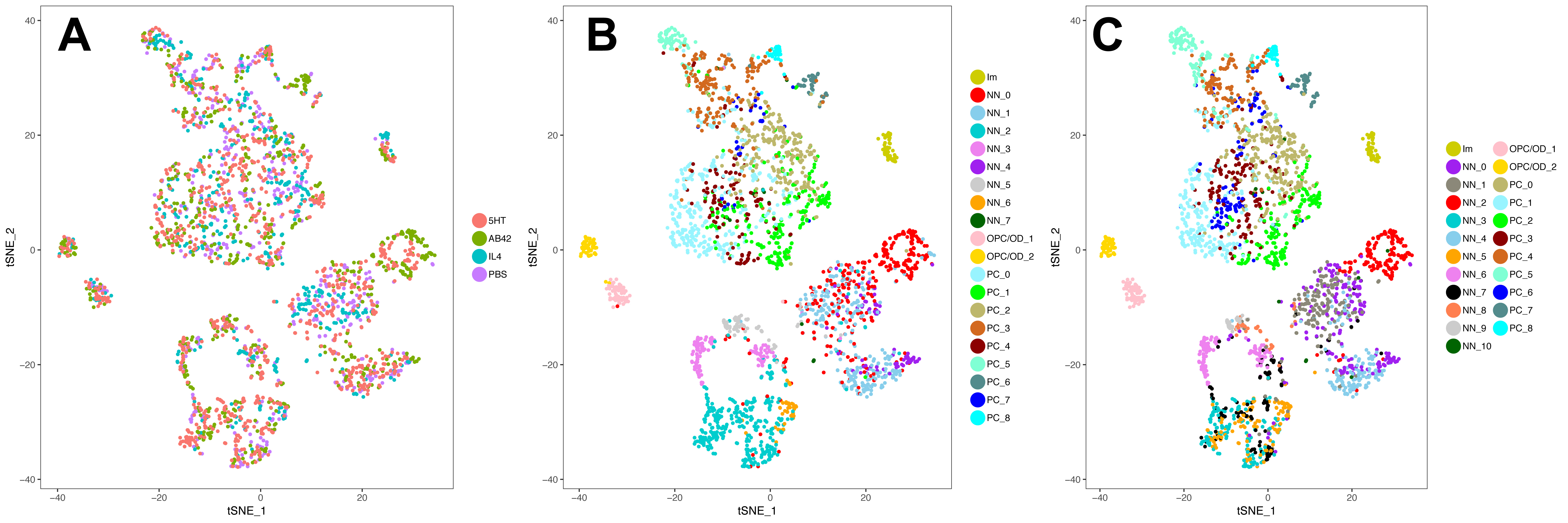

Supplement: S5 Fig — Cells are color-coded in samples (A), cell clusters predicted by RandomForest (B), and cell clusters identified by Seurat (C) after using all 4 experimental groups together. To use the same neuronal and progenitor clusters we identified before ([34]), we used RandomForest and machine learning (B) in our analyses. By using Seurat (C), cell clusters can also be inferred de novo. The cell clusters and their top marker genes are identical, whereas some cell clusters (e.g., neurons) can be further subdivided depending on the algorithm used. The color codes used in the middle panel are the same colors used in [34]. The colors of PCs are also used in Seurat analyses (A). A few cells from Aβ42 and 5-HT groups do not exist in other groups (control and IL4). These cells express olfactory bulb markers and are contaminations of cells in sample preparation. They cluster separately from all groups we analyzed and are not affecting the biological outcomes of the analyses. Related to Fig 3. See S3 Data for supporting information. Aβ42, amyloid-beta42; IL4, interleukin-4; PC, progenitor cell; 5-HT, serotonin. (JPG) [file pbio.3000585.s005.jpg]

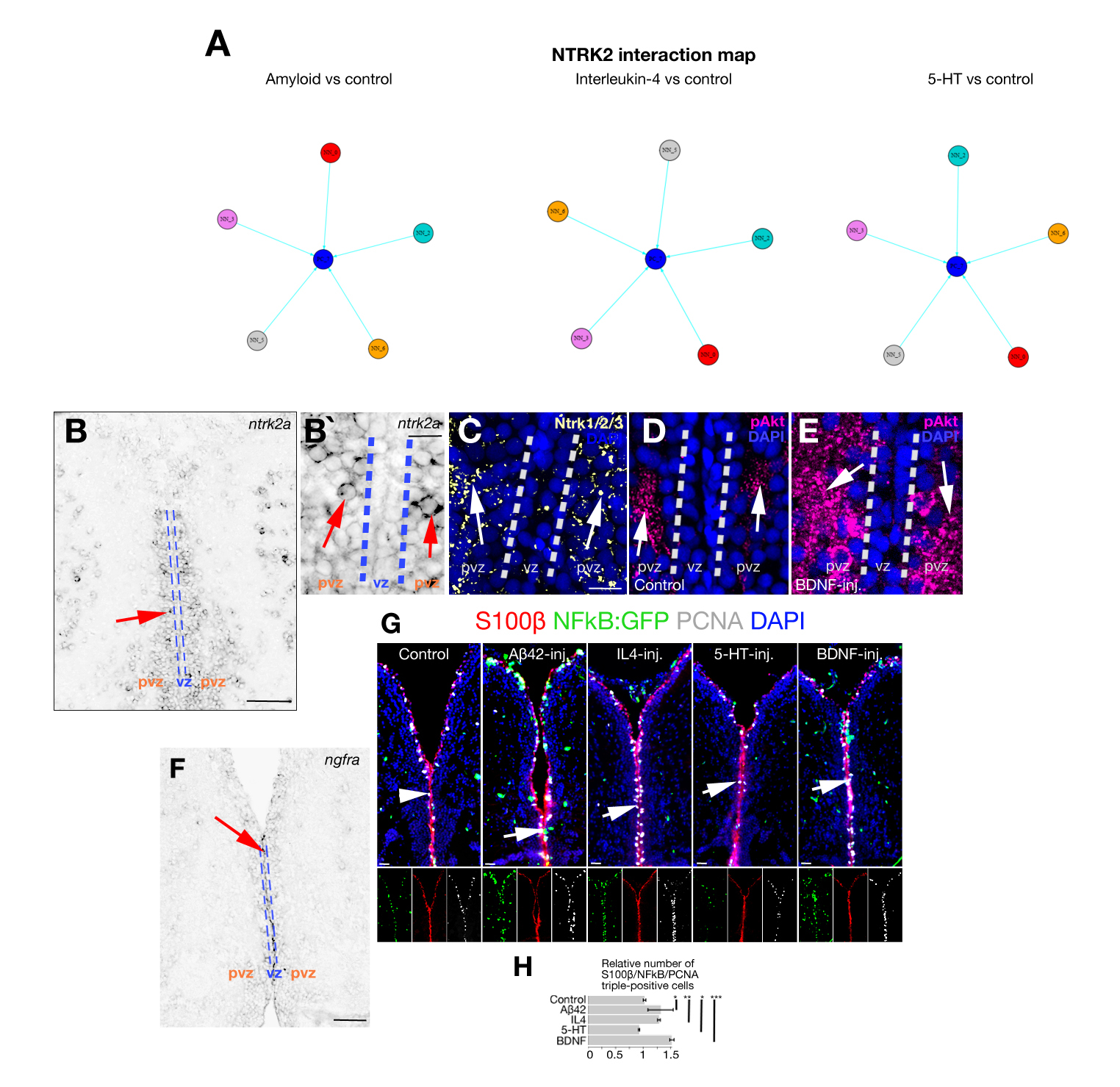

Supplement: S6 Fig — (A) In silico interaction map for NTRK2 in Aβ42 versus control, IL4 versus control, and 5-HT versus control comparisons. Black arrows: interactions unchanged with treatment, cyan arrows: interaction lost with treatment, magenta arrows: interaction gained/emerged with the treatment. (B) ISH for ntrk2 in zebrafish brain. (Bʹ) Close-up image. Note the expression in pvz but not in vz that contains the NSCs. (C) IHC for Ntrk2 protein in zebrafish brain, supporting the ISH results and presence of Ntrk2 in pvz. (D, E) IHC for pAkt in control (D) and BDNF-injected (E) brains. BDNF activates pAkt in pvz but not in vz. (F) ISH for ngfra in adult zebrafish telecephalon. (G) IHC for S100β, NfkB-driven GFP, and PCNA in control, Amyloid-injected, IL4-injected, 5-HT-injected, and BDNF-injected brains. Smaller panels under larger images show individual fluorescent channels. (H) Quantification of the relative number of proliferating NSCs that have active NFkB signaling. Scale bars equal 100 μM. Data are represented as mean ± SEM. Related to Fig 4. See S7 Data for supporting information. BDNF, brain-derived neurotrophic factor; GFP, green fluorescent protein; IHC, immunohistochemistry; IL4, interleukin-4; ISH, in situ hybridization; NFkB, nuclear factor 'kappa-light-chain-enhancer' of activated B-cells; NSC, neural stem cell; NTRK2, neurotrophic tyrosine kinase receptor, type 2; pAkt, phosphorylated protein kinase B; PCNA, proliferating cell nuclear antigen; pvz, periventricular zone; S100β, S100 calcium-binding protein B; vz, ventricular zone; 5-HT, serotonin. (JPG) [file pbio.3000585.s006.jpg]

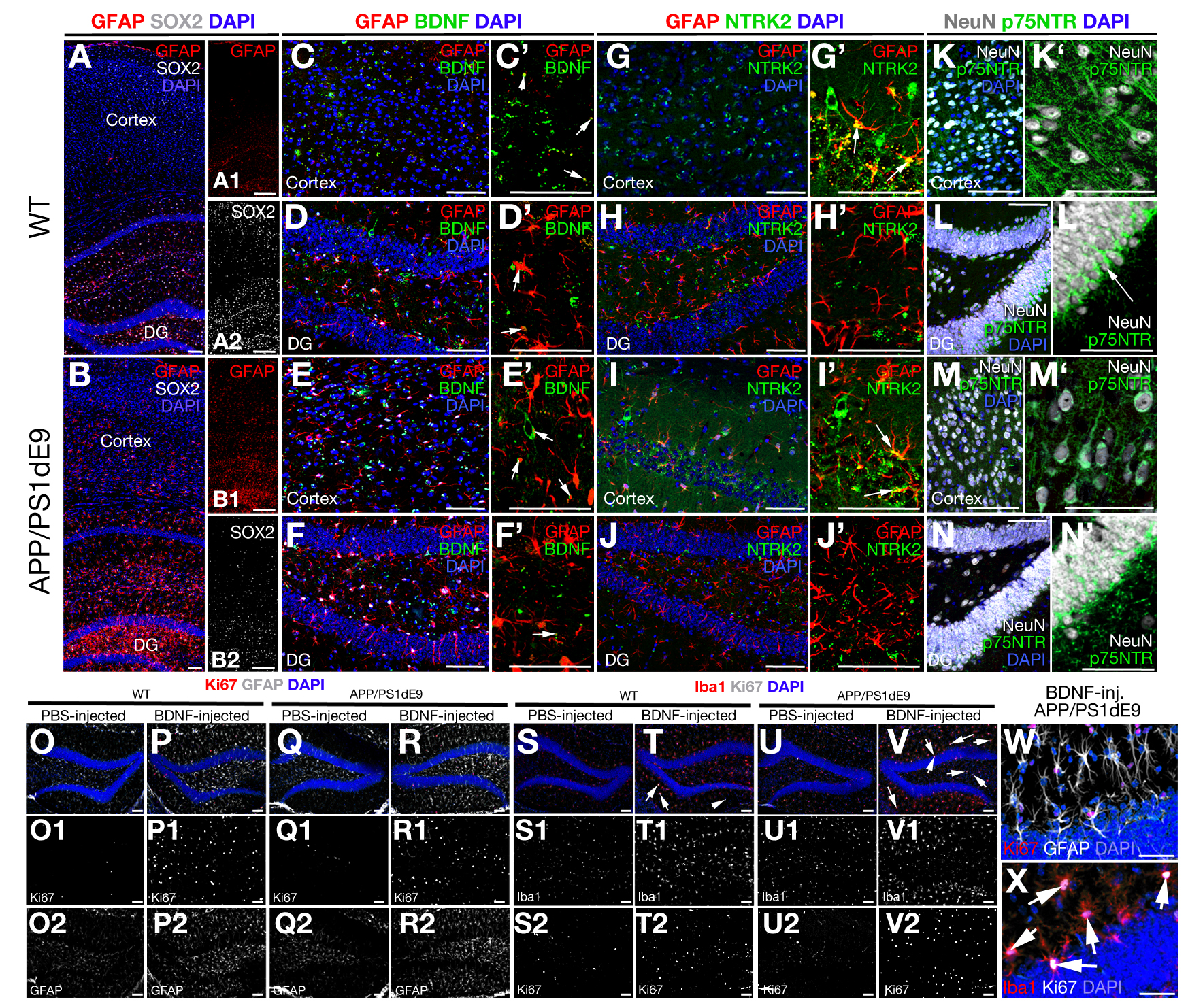

Supplement: S7 Fig — (A, B) IHC for GFAP and SOX2 in WT (A) and APP/PS1dE9 mouse brains (B) at 12 months of age. (A1, A2) Single fluorescent channels of panel A. (B1, B2) Single fluorescent channels of panel B. (C–Dʹ) IHC for GFAP and BDNF in WT mouse: (C) cortex, (D) DG. Primed images are higher magnification without DAPI. (E–Fʹ) IHC for GFAP and BDNF in APP/PS1dE9 mouse: (E) cortex, (F) DG. Primed images are higher magnification without DAPI. (G–Hʹ) IHC for GFAP and NTRK2 in WT mouse: (G) cortex, (H) DG. Primed images are higher magnification without DAPI. (I–Jʹ) IHC for GFAP and NTRK2 in APP/PS1dE9 mouse: (I) cortex, (J) DG. Primed images are higher magnification without DAPI. (K–Lʹ) IHC for NeuN and p75/NTR in WT mouse: (K) cortex, (L) DG. Primed images are higher magnification without DAPI. (M–Nʹ) IHC for NeuN and p75/NTR in APP/PS1dE9 mouse: (M) cortex, (N) DG. Primed images are higher magnification without DAPI. (O–R2) IHC for Ki67 and GFAP in WT mouse PBS-injected hemisphere (O), wt mouse BDNF-injected hemisphere (P), APP/PS1dE9 mouse PBS-injected hemisphere (Q), APP/PS1dE9 mouse BDNF-injected hemisphere (R). (S–V2) IHC for Ki67 and Iba1 in WT mouse PBS-injected hemisphere (S), WT mouse BDNF-injected hemisphere (T), APP/PS1dE9 mouse PBS-injected hemisphere (U), APP/PS1dE9 mouse BDNF-injected hemisphere (V). (W) High-magnification image of Ki67 and GFAP staining on DG of BDNF-injected hemisphere of APP/PS1dE9 mouse. GFAP cells do not proliferate after BDNF injection. (X) High-magnification image of Ki67 and Iba1 staining on DG of BDNF-injected hemisphere of APP/PS1dE9 mouse. Most Ki67-positive cells overlap with Iba1 staining after BDNF injection. n = 2 animals used for analyses. Scale bars equal 100 μM. Related to Fig 4. AD, Alzheimer disease; BDNF, brain-derived neurotrophic factor; DG, dentate gyrus; GFAP, glial fibrillary acidic protein; Iba1, ionized calcium binding adaptor molecule 1; IHC, immunohistochemistry; Ki67, antigen identified by monoclonal antibody Ki-67; NeuN, Fo [file pbio.3000585.s007.jpg]

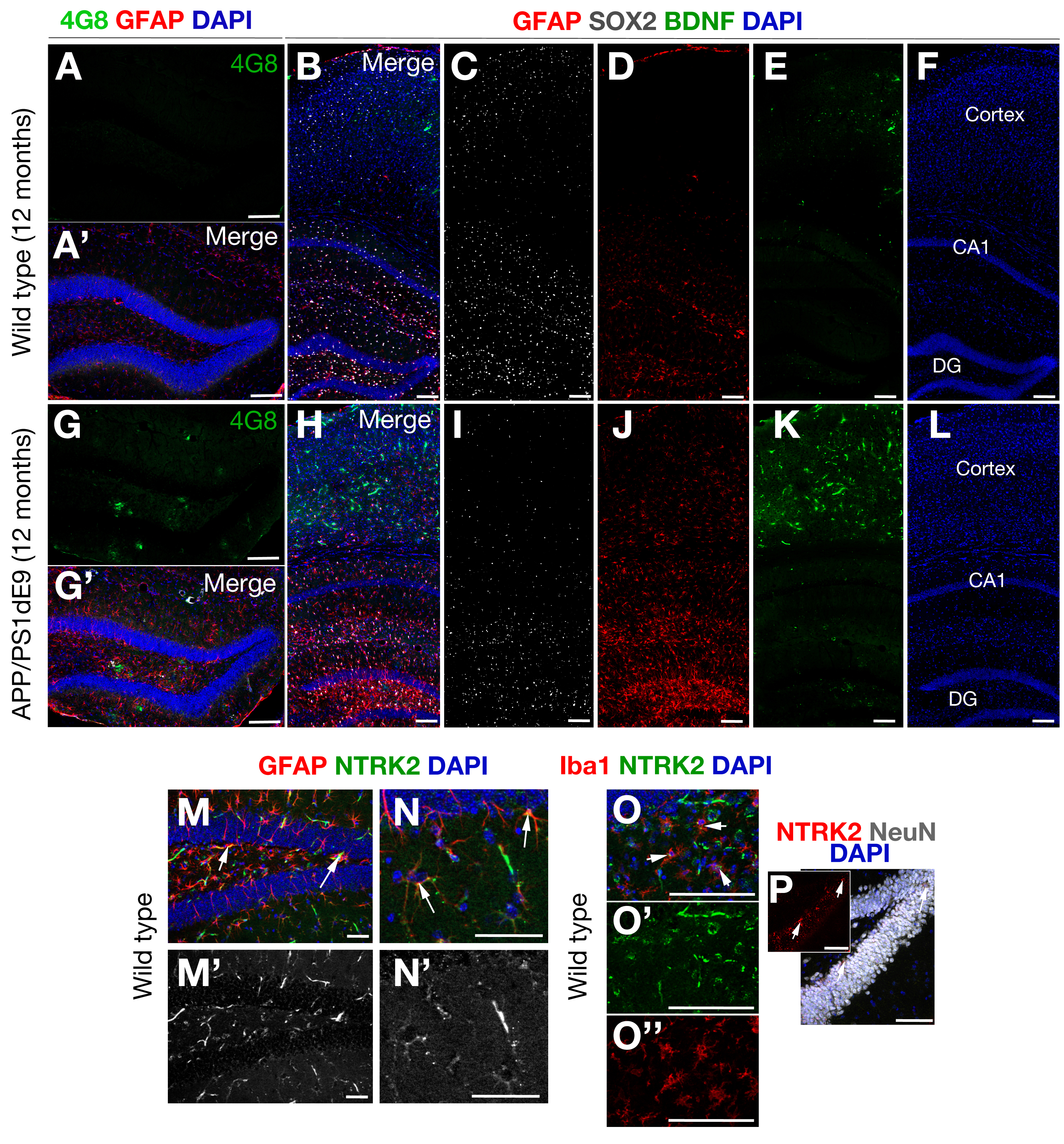

Supplement: S8 Fig — (A, Aʹ) IHC for amyloid plaques (4G8) in wild-type mouse hippocampus. (B–F) IHC for GFAP, SOX2, and BDNF in wild-type mouse brains. (G, Gʹ) IHC for amyloid plaques (4G8) in APP/PS1dE9 mouse hippocampus. (H–L) IHC for GFAP, SOX2, and BDNF in APP/PS1dE9 mouse brains. Mice were at the age of 12 months. (M) IHC for GFAP and NTRK2 in wild-type mouse DG. (Mʹ) Single fluorescent channel for NTRK2. (N) High magnification of merged image. (Nʹ) Single fluorescent channel for NTRK2 in panel N. (O) IHC for Iba1 and NTRK2. (Oʹ) Single fluorescent channel for NTRK2 in panel O. (Oʺ) single fluorescent channel for Iba1 in panel O. (P) IHC for NTRK2 and NeuN. Single channel in red is NTRK2. Scale bars equal 100 μM. Related to Figs 4 and 5. BDNF, brain-derived neurotrophic factor; DG, dentate gyrus; GFAP, glial fibrillary acidic protein; Iba1, ionized calcium binding adaptor molecule 1; IHC, immunohistochemistry; NeuN, Fox-3, Rbfox3, or Hexaribonucleotide binding protein 3; IHC, immunohistochemistry;; NTRK2, neurotrophic tyrosine kinase, receptor, type 2; SOX2, (sex determining region Y)-box transcription factor 2. (JPG) [file pbio.3000585.s008.jpg]

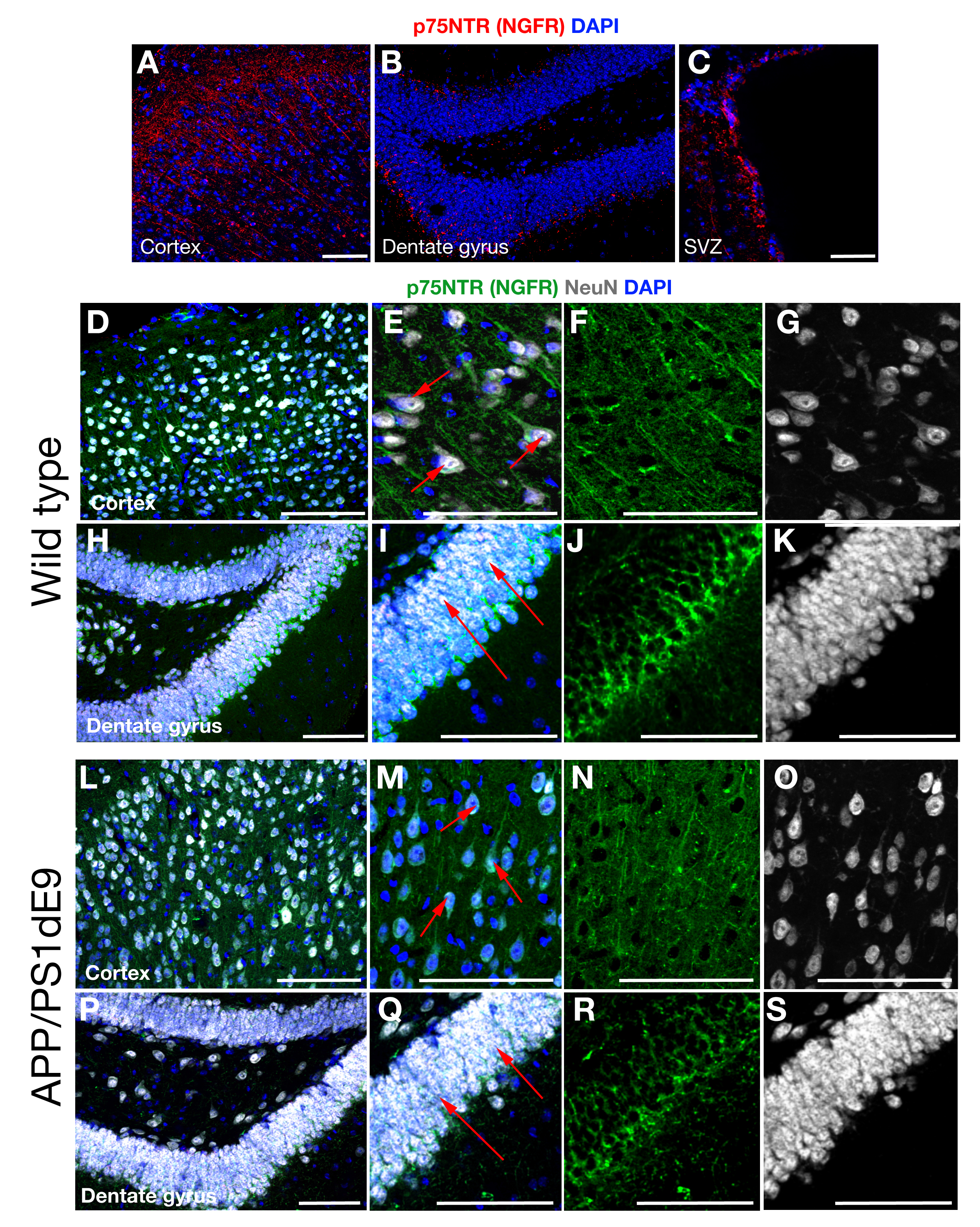

Supplement: S9 Fig — (A–C) IHC for p75/NTR (NGFR) in wild-type cortex (A), DG (B), and SVZ (C). (D–K) IHC for p75/NTR and NeuN in cortex (D) and DG (H). (E–G) High-magnification image from panel D. (I–K) High-magnification image from panel H. (L–S) IHC for p75/NTR and NeuN in cortex (L) and DG (P) of APP/PS1dE9 mouse. (M–O) High-magnification image from panel L. (Q–S) High-magnification image from panel P. Scale bars equal 100 μM. Related to Figs 4 and 5. DG, dentate gyrus; IHC, immunohistochemistry; NeuN, Fox-3, Rbfox3, or Hexaribonucleotide binding protein 3; NGFR, nerve growth factor receptor; p75/NTR, neurotrophin receptor P75; SVZ, subventricular zone. (JPG) [file pbio.3000585.s009.jpg]

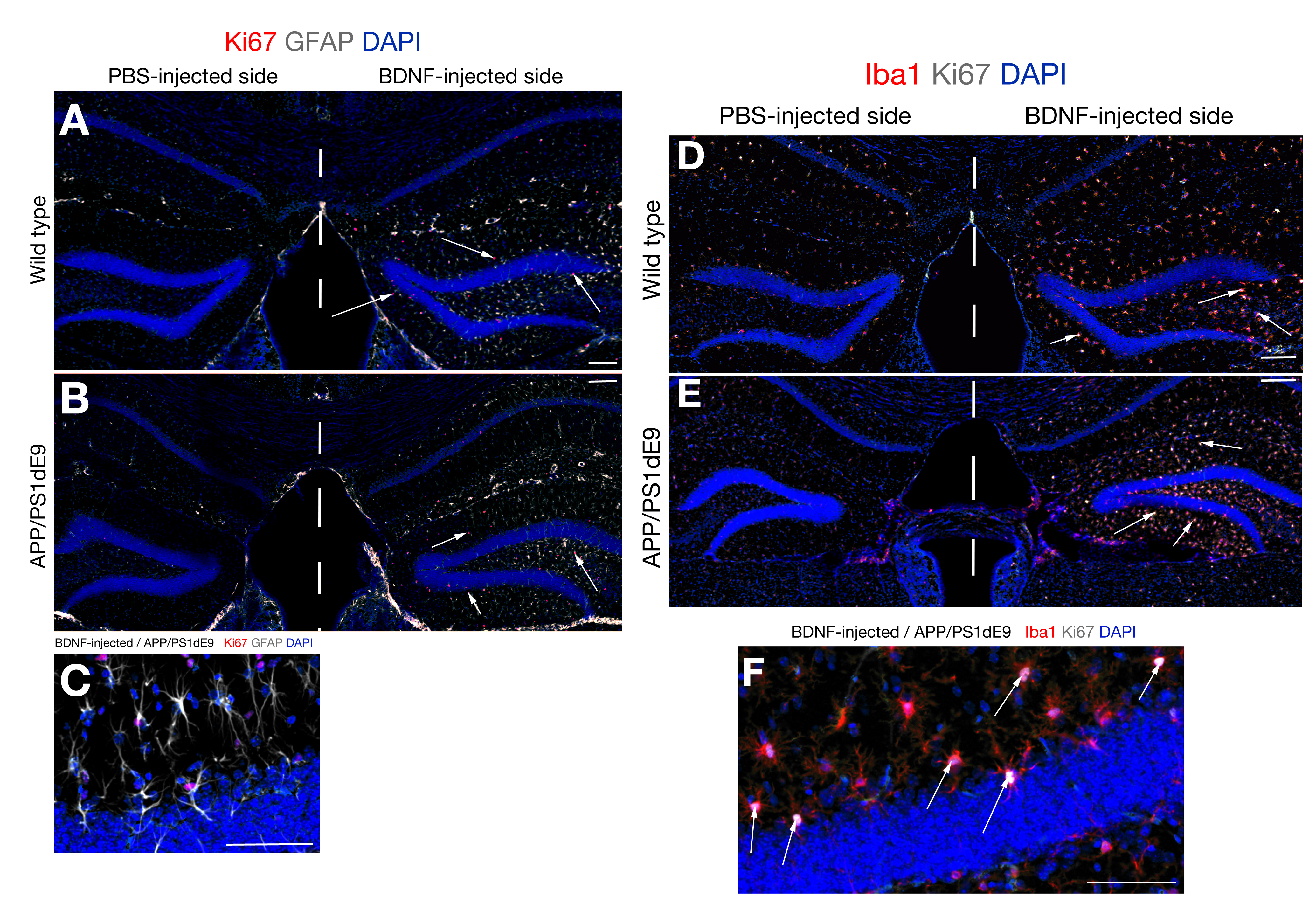

Supplement: S10 Fig — (A, B) IHC for Ki67 and GFAP in wild-type (A) and APP/PS1dE9 (B) mouse brains at 12 months of age after injection of PBS (left hemisphere) and BDNF (right hemisphere). (C) Close-up image of the DG of a BDNF-injected side of the APP/PS1dE9 mouse brain. (D, E) IHC for Ki67 and Iba1 in wild-type (D) and APP/PS1dE9 (E) mouse brains at 12 months of age after injection of PBS (left hemisphere) and BDNF (right hemisphere). (F) Close-up image of the DG of a BDNF-injected side of the APP/PS1dE9 mouse brain. Scale bars equal 100 μM. Related to Figs 4 and 5. BDNF, brain-derived neurotrophic factor; DG, dentate gyrus; Ki67, antigen identified by monoclonal antibody Ki-67; GFAP, glial fibrillary acidic protein; Iba1, ionized calcium binding adaptor molecule 1; IHC, immunohistochemistry; NeuN, Fox-3, Rbfox3, or Hexaribonucleotide binding protein 3; IHC, immunohistochemistry. (JPG) [file pbio.3000585.s010.jpg]
