# Supplementary material for: Neuron-glia interaction through Serotonin-BDNF-NGFR axis enables regenerative neurogenesis in Alzheimer’s model of adult zebrafish brain
Source: PLoS Biol. 2020 Jan 6;18(1):e3000585. doi: 10.1371/journal.pbio.3000585 (PMC6964913; doi:10.1371/journal.pbio.3000585)
Supplement: S3 Data — tSNE, t-Distributed stochastic neighbor embedding; VLN, violin plot. (PDF) [file pbio.3000585.s013.pdf]

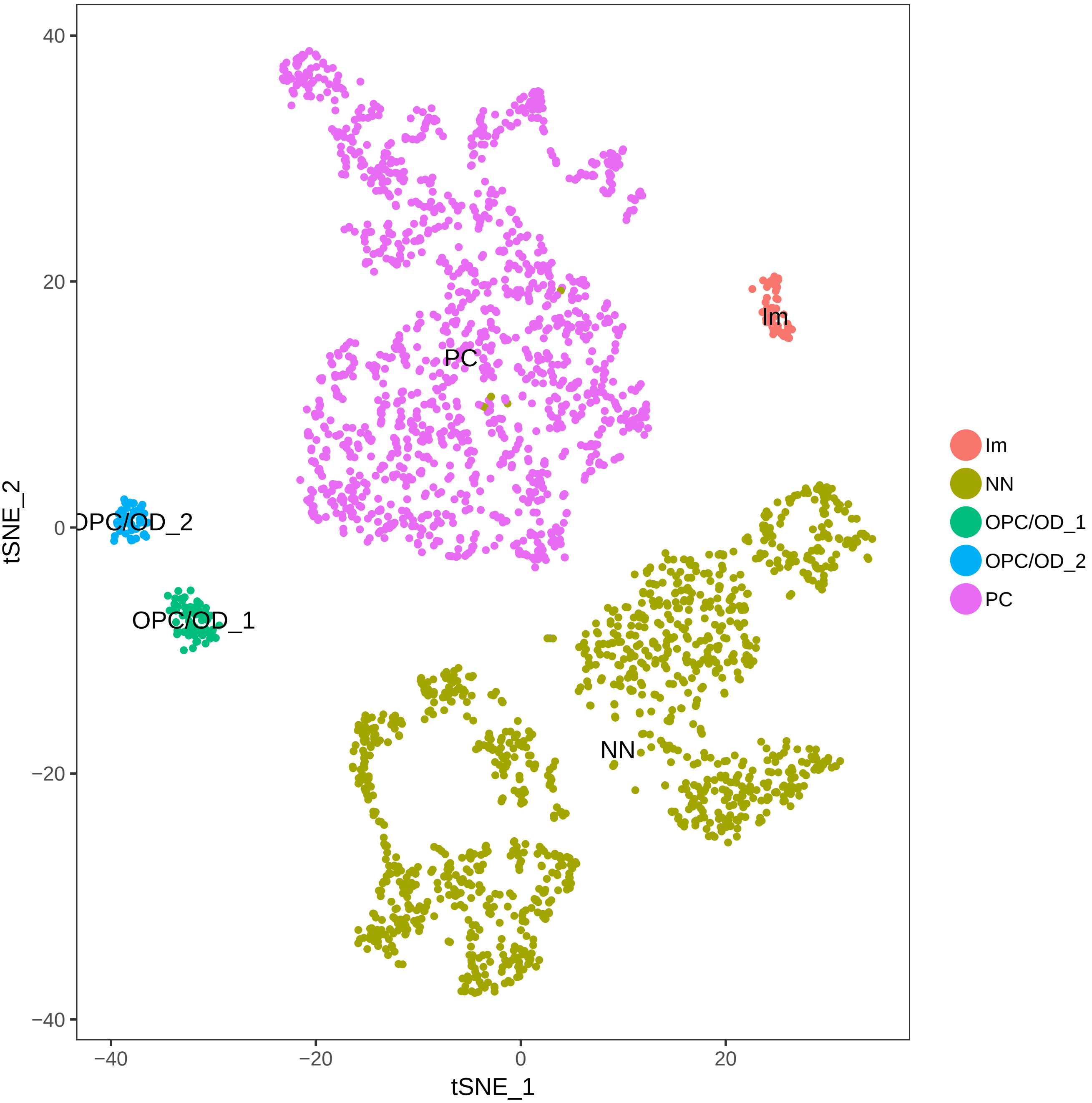

PC
Im
OPC/OD_2
OPC/OD_1
NN
tSNE_1
tSNE_2
40
20
0
−20
−40
−40
−20
0
20
Im
NN
OPC/OD_1
OPC/OD_2
PC

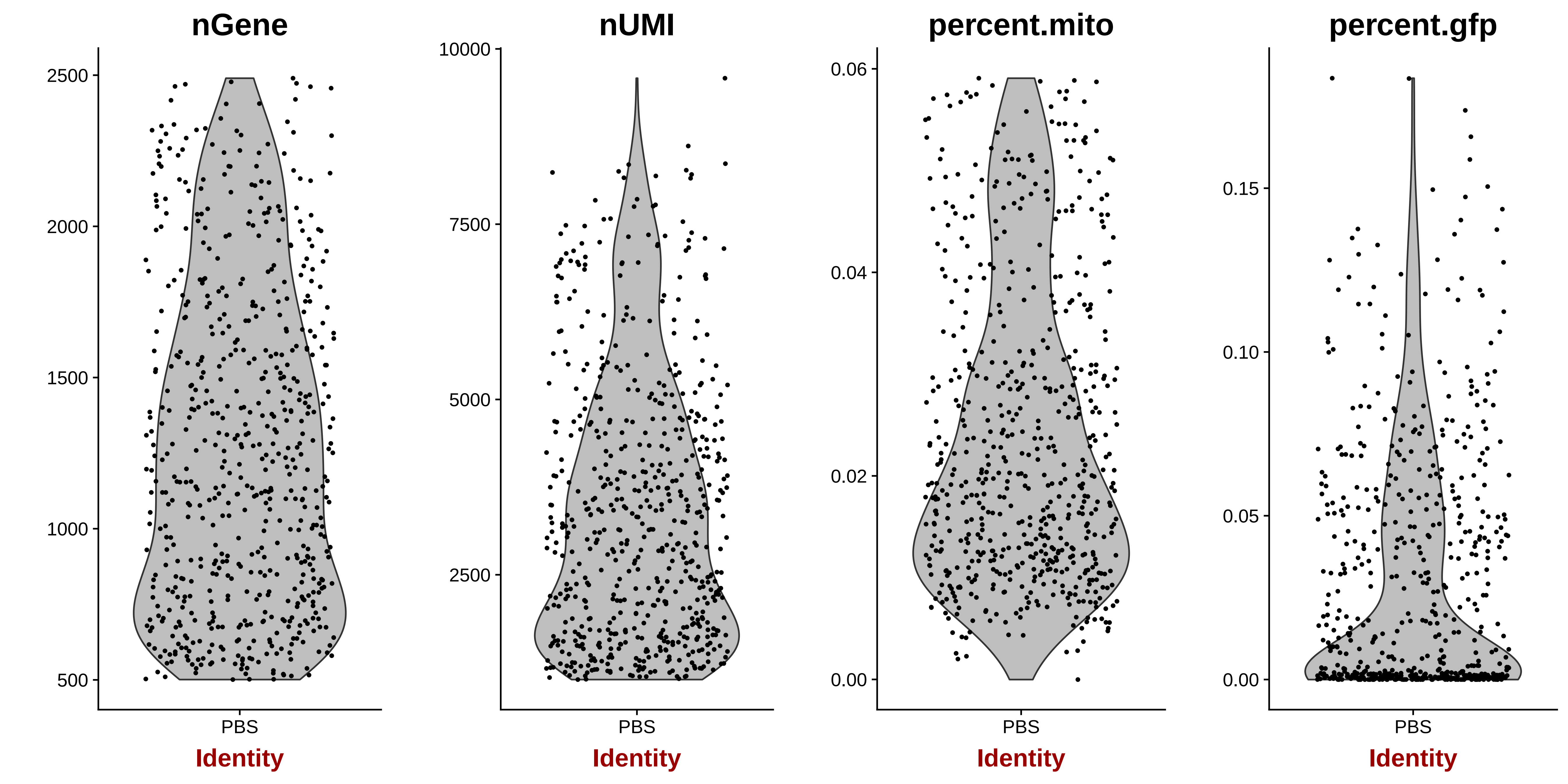
nGene
2500
2000
1500
1000
500
PBS
Identity
nUMI
10000
7500
5000
2500
PBS
Identity
percent.mito
0.06
0.04
0.02
0.00
PBS
Identity
percent.gfp
0.15
0.10
0.05
0.00
PBS
Identity

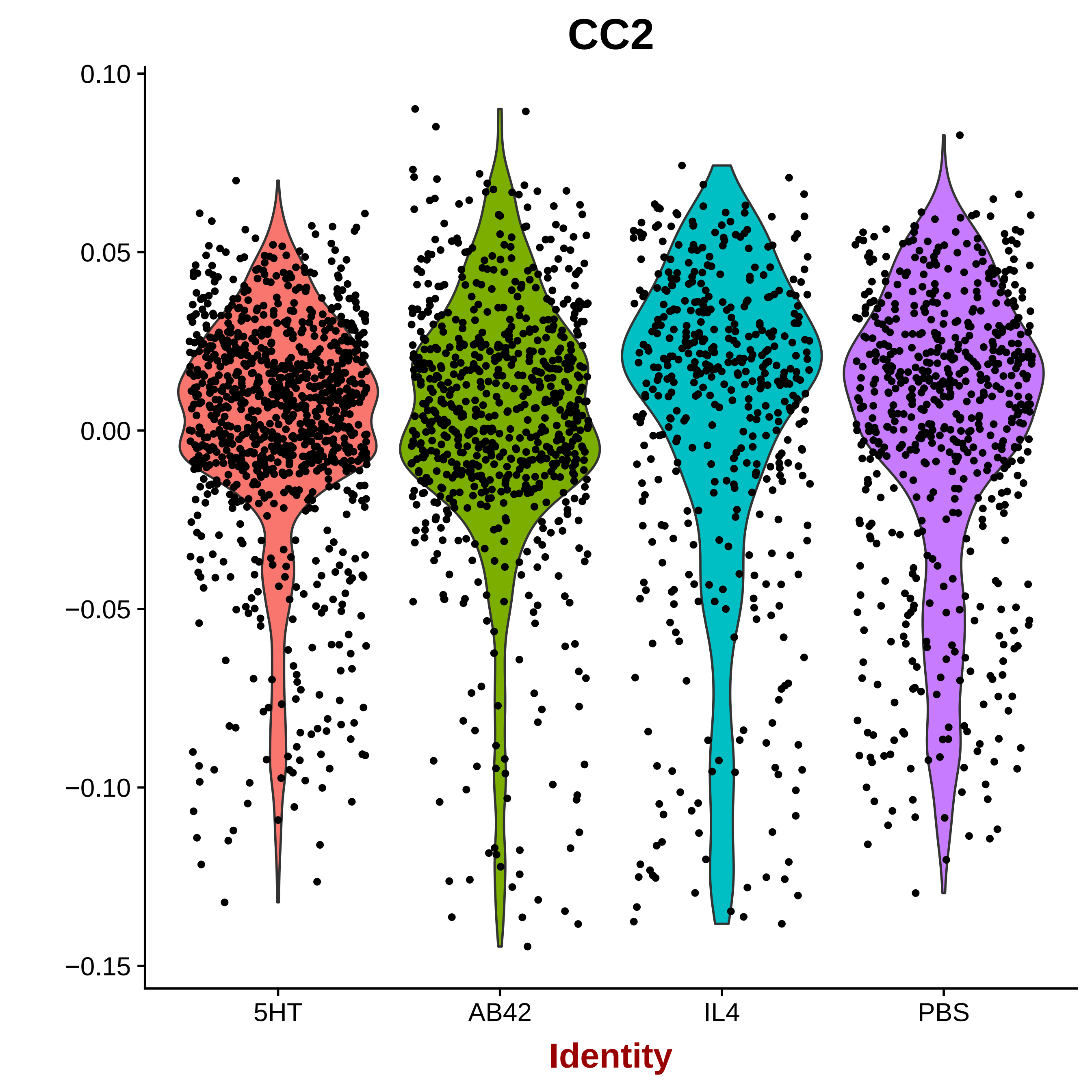
CC2
0.10
0.05
0.00
−0.05
−0.10
−0.15
5HT
AB42
IL4
PBS
Identity

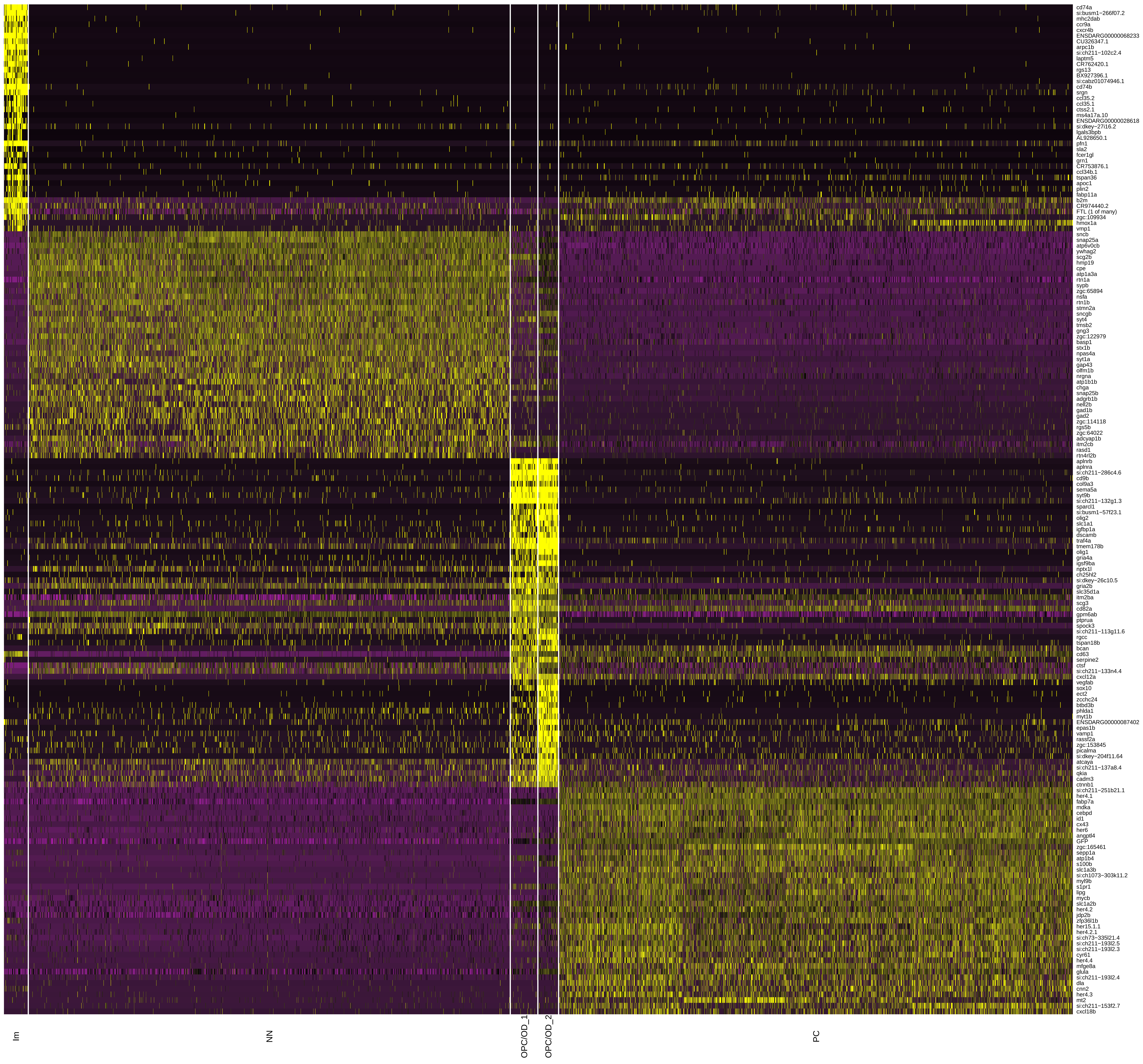
cd74a
si:busm1−266f07.2
mhc2dab
ccr9a
cxcr4b
ENSDARG00000068233
CU326347.1
arpc1b
si:ch211−102c2.4
laptm5
CR762420.1
rgs13
BX927396.1
si:cabz01074946.1
cd74b
srgn
ccl35.2
ccl35.1
ctss2.1
ms4a17a.10
ENSDARG00000028618
si:dkey−27i16.2
lgals3bpb
AL928650.1
pfn1
sla2
fcer1gl
grn1
CR753876.1
ccl34b.1
tspan36
apoc1
plin2
fabp11a
b2m
CR974440.2
FTL (1 of many)
zgc:109934
hmox1a
vmp1
sncb
snap25a
atp6v0cb
ywhag2
scg2b
hmp19
cpe
atp1a3a
rtn1a
sypb
zgc:65894
nsfa
rtn1b
stmn2a
sncgb
syt4
tmsb2
gng3
zgc:122979
basp1
stx1b
npas4a
syt1a
gap43
olfm1b
nrgna
atp1b1b
chga
snap25b
adgrb1b
nell2b
gad1b
gad2
zgc:114118
rgs5b
zgc:64022
adcyap1b
itm2cb
rasd1
rtn4rl2b
aplnrb
aplnra
si:ch211−286c4.6
cd9b
col9a3
sema5a
syt9b
si:ch211−132g1.3
sparcl1
si:busm1−57f23.1
olig2
slc1a1
igfbp1a
dscamb
traf4a
tmem178b
olig1
gria4a
igsf9ba
nptx1l
ch25hl2
si:dkey−26c10.5
gria2b
slc35d1a
itm2ba
scg3
cd82a
gpm6ab
ptprua
spock3
si:ch211−113g11.6
rgcc
tspan18b
bcan
cd63
serpine2
ctsf
si:ch211−133n4.4
cxcl12a
vegfab
sox10
ect2
zcchc24
btbd3b
phlda1
myt1b
ENSDARG00000087402
epas1b
vamp1
rassf2a
zgc:153845
picalma
si:dkey−204f11.64
atcaya
si:ch211−137a8.4
qkia
cadm3
ctnnb1
si:ch211−251b21.1
her4.1
fabp7a
mdka
cebpd
id1
cx43
her6
angptl4
GFP
zgc:165461
sepp1a
atp1b4
s100b
slc1a3b
si:ch1073−303k11.2
myl9b
s1pr1
lipg
mycb
slc1a2b
her4.2
jdp2b
zfp36l1b
her15.1.1
her4.2.1
si:ch73−335l21.4
si:ch211−193l2.5
si:ch211−193l2.3
cyr61
her4.4
mfge8a
glula
si:ch211−193l2.4
dla
cnn2
her4.3
mt2
si:ch211−153f2.7
cxcl18b
Im
NN
OPC/OD_1
OPC/OD_2
PC

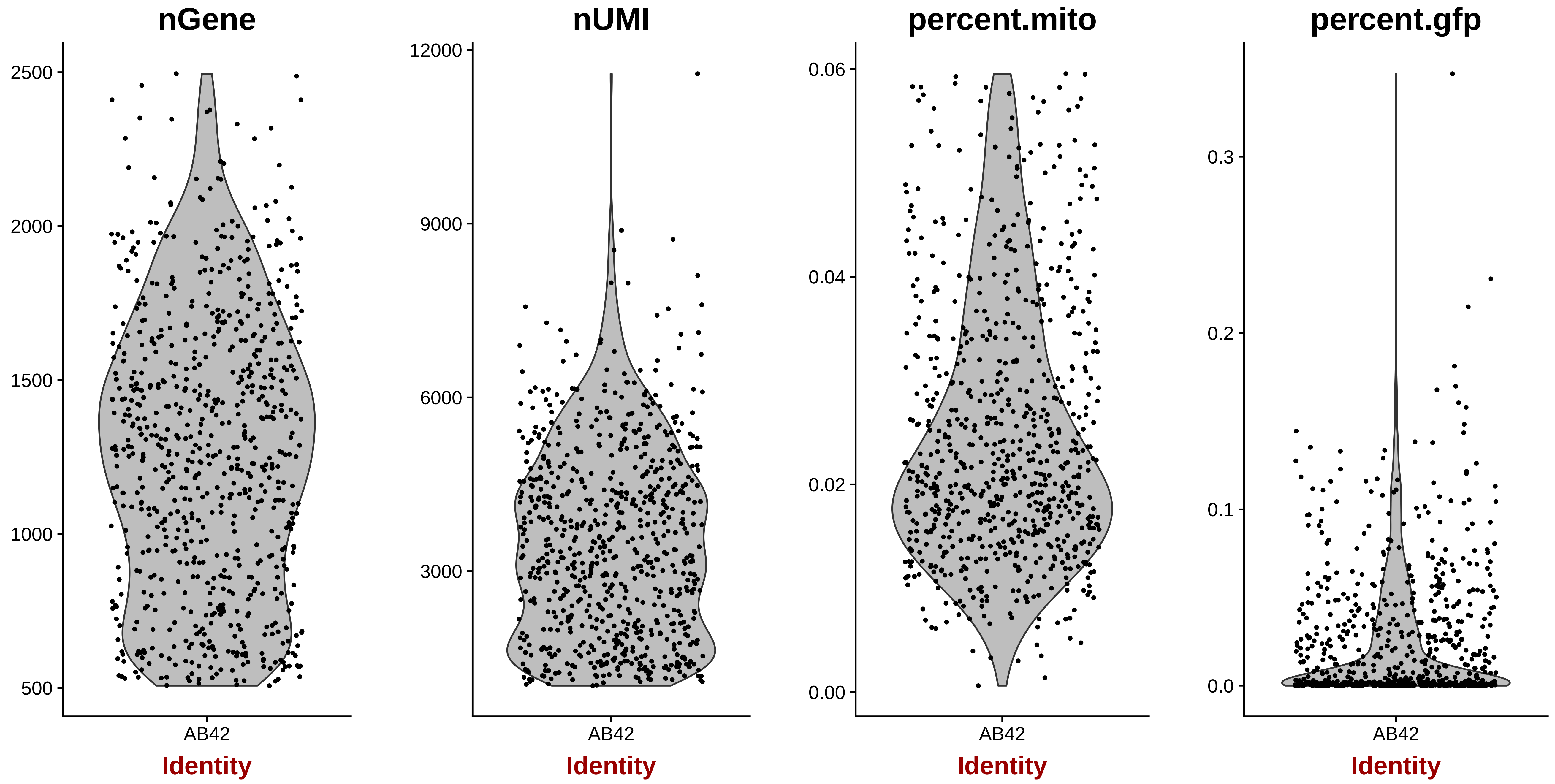

nGene
nUMI
percent.mito
percent.gfp
2500
2000
1500
1000
500
12000
9000
6000
3000
0.06
0.04
0.02
0.00
0.3
0.2
0.1
0.0
AB42
Identity

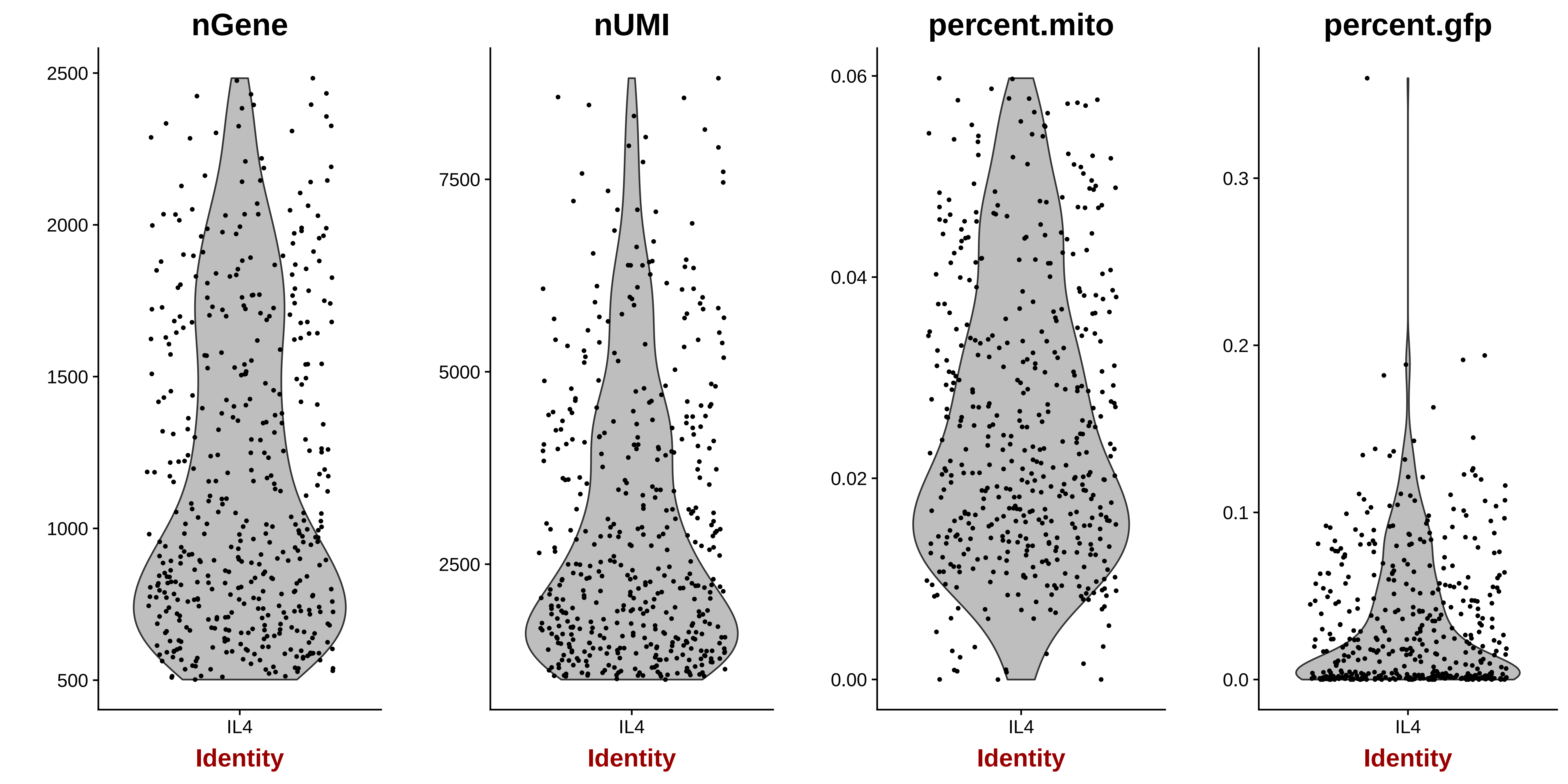

nGene
nUMI
percent.mito
percent.gfp
2500
2000
1500
1000
500
7500
5000
2500
0.06
0.04
0.02
0.00
0.3
0.2
0.1
0.0
IL4
Identity

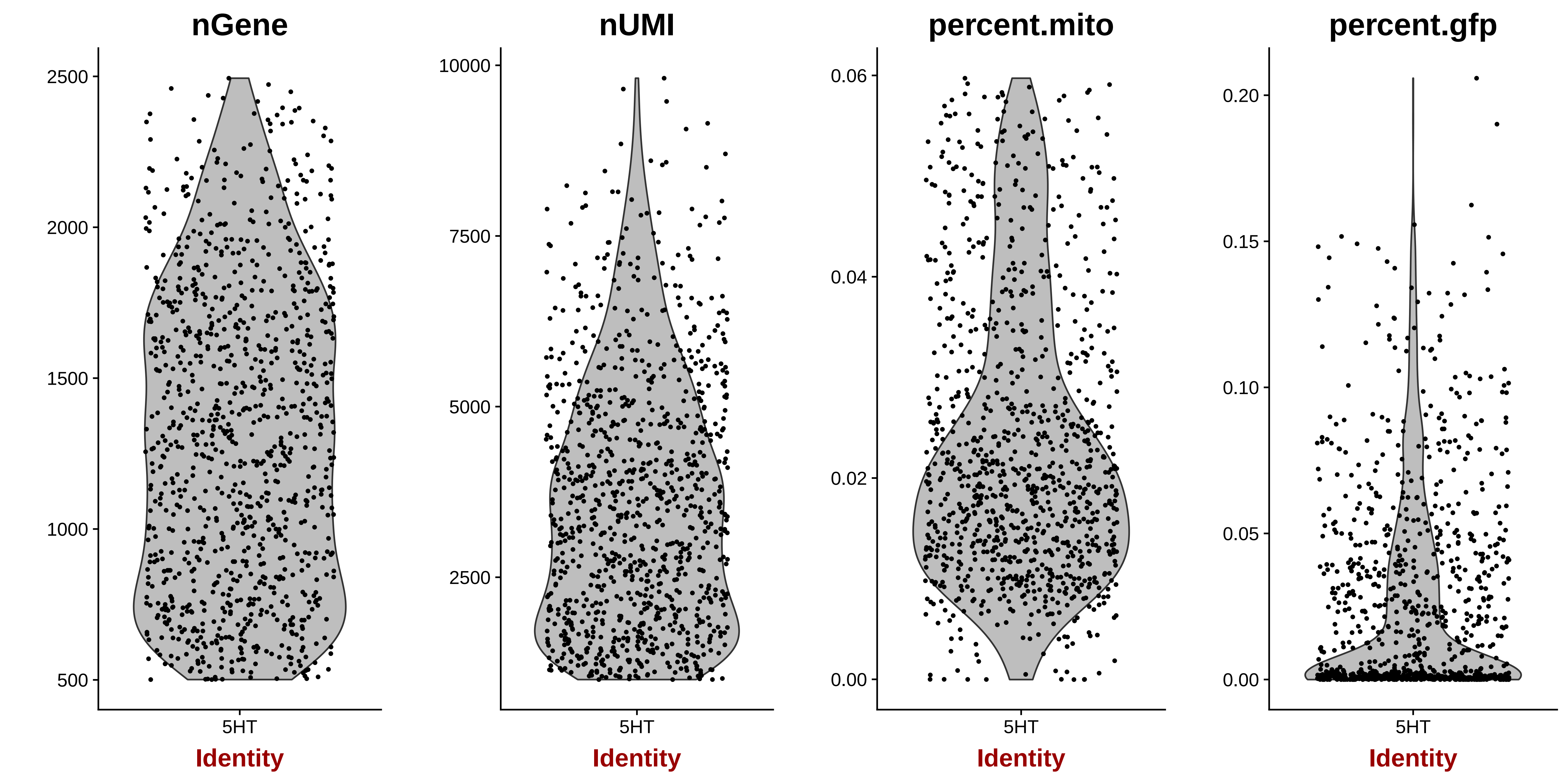

nGene
nUMI
percent.mito
percent.gfp
2500
2000
1500
1000
500
10000
7500
5000
2500
0.06
0.04
0.02
0.00
0.20
0.15
0.10
0.05
0.00
5HT
5HT
5HT
5HT
Identity
Identity
Identity
Identity

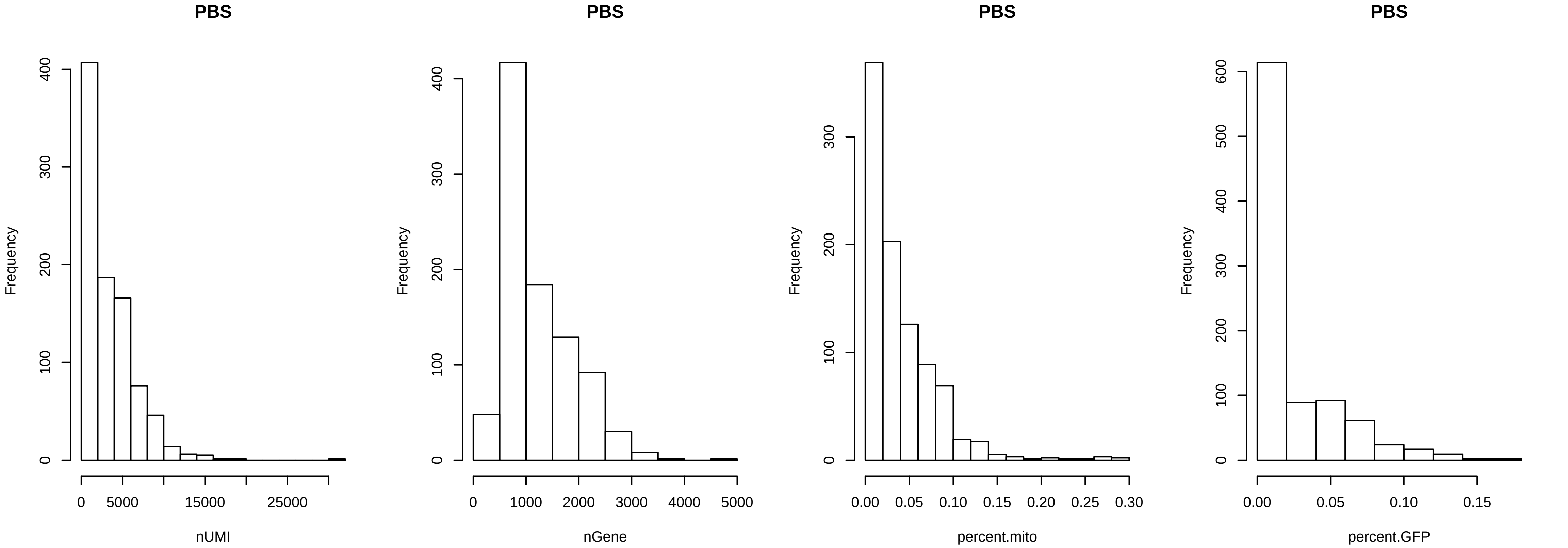
PBS
Frequency
nUMI
0
5000
15000
25000
0
100
200
300
400
PBS
Frequency
nGene
0
1000
2000
3000
4000
5000
0
100
200
300
400
PBS
Frequency
percent.mito
0.00
0.05
0.10
0.15
0.20
0.25
0.30
0
100
200
300
PBS
Frequency
percent.GFP
0.00
0.05
0.10
0.15
0
100
200
300
400
500
600

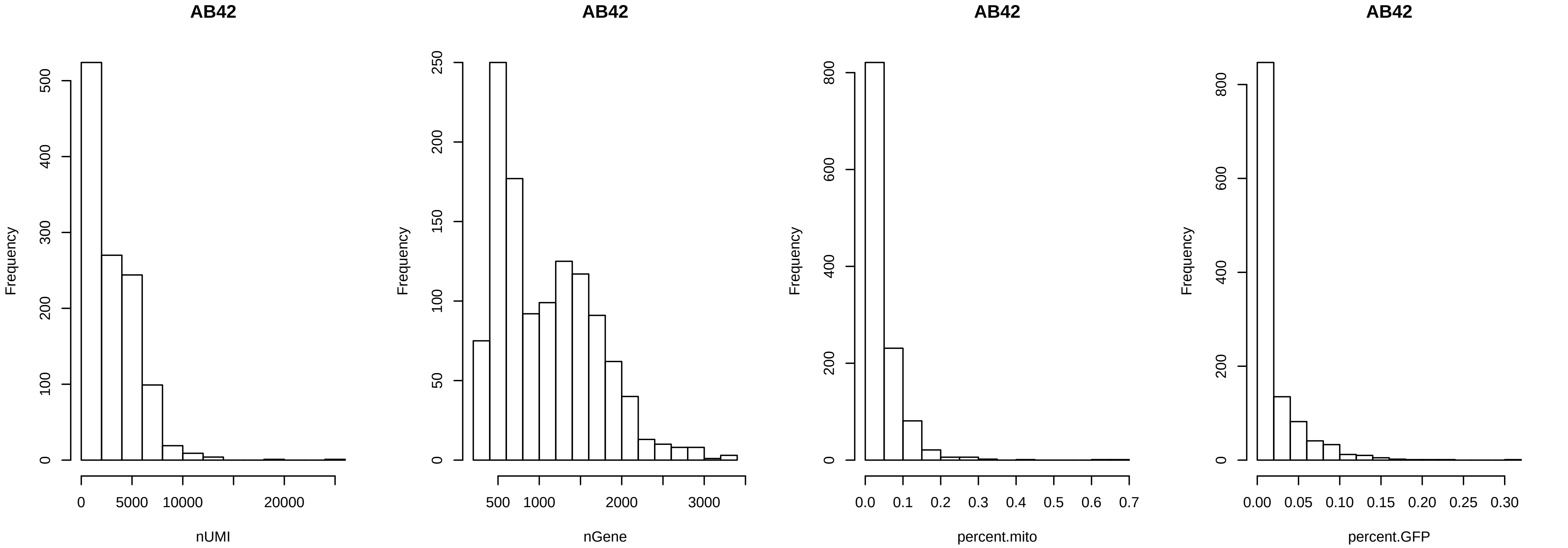
AB42
Frequency
0
100
200
300
400
500
0
5000
10000
20000
nUMI
AB42
Frequency
0
50
100
150
200
250
500
1000
2000
3000
nGene
AB42
Frequency
0
200
400
600
800
0.0
0.1
0.2
0.3
0.4
0.5
0.6
0.7
percent.mito
AB42
Frequency
0
200
400
600
800
0.00
0.05
0.10
0.15
0.20
0.25
0.30
percent.GFP

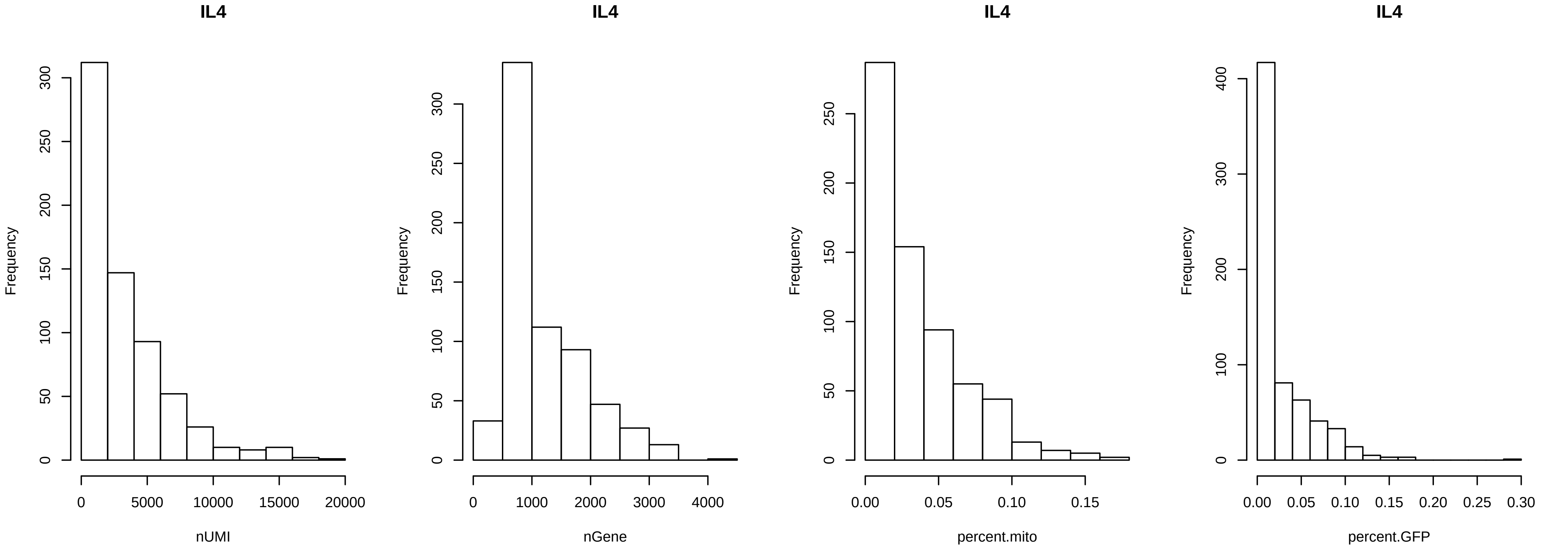

IL4
Frequency
0
50
100
150
200
250
300
0
5000
10000
15000
20000
nUMI
IL4
Frequency
0
50
100
150
200
250
300
0
1000
2000
3000
4000
nGene
IL4
Frequency
0
50
100
150
200
250
0.00
0.05
0.10
0.15
percent.mito
IL4
Frequency
0
100
200
300
400
0.00
0.05
0.10
0.15
0.20
0.25
0.30
percent.GFP

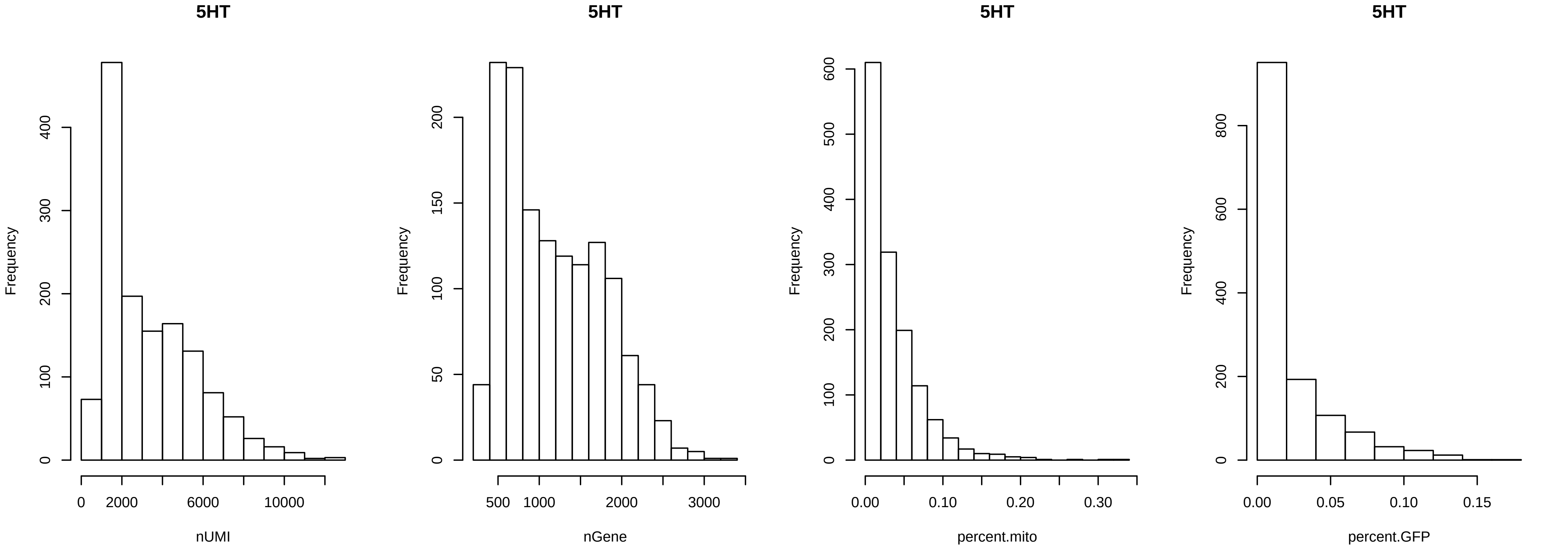

5HT
Frequency
nUMI
0
100
200
300
400
2000
6000
10000
5HT
Frequency
nGene
0
50
100
150
200
500
1000
2000
3000
5HT
Frequency
percent.mito
0
100
200
300
400
500
600
0.00
0.10
0.20
0.30
5HT
Frequency
percent.GFP
0
200
400
600
800
0.00
0.05
0.10
0.15

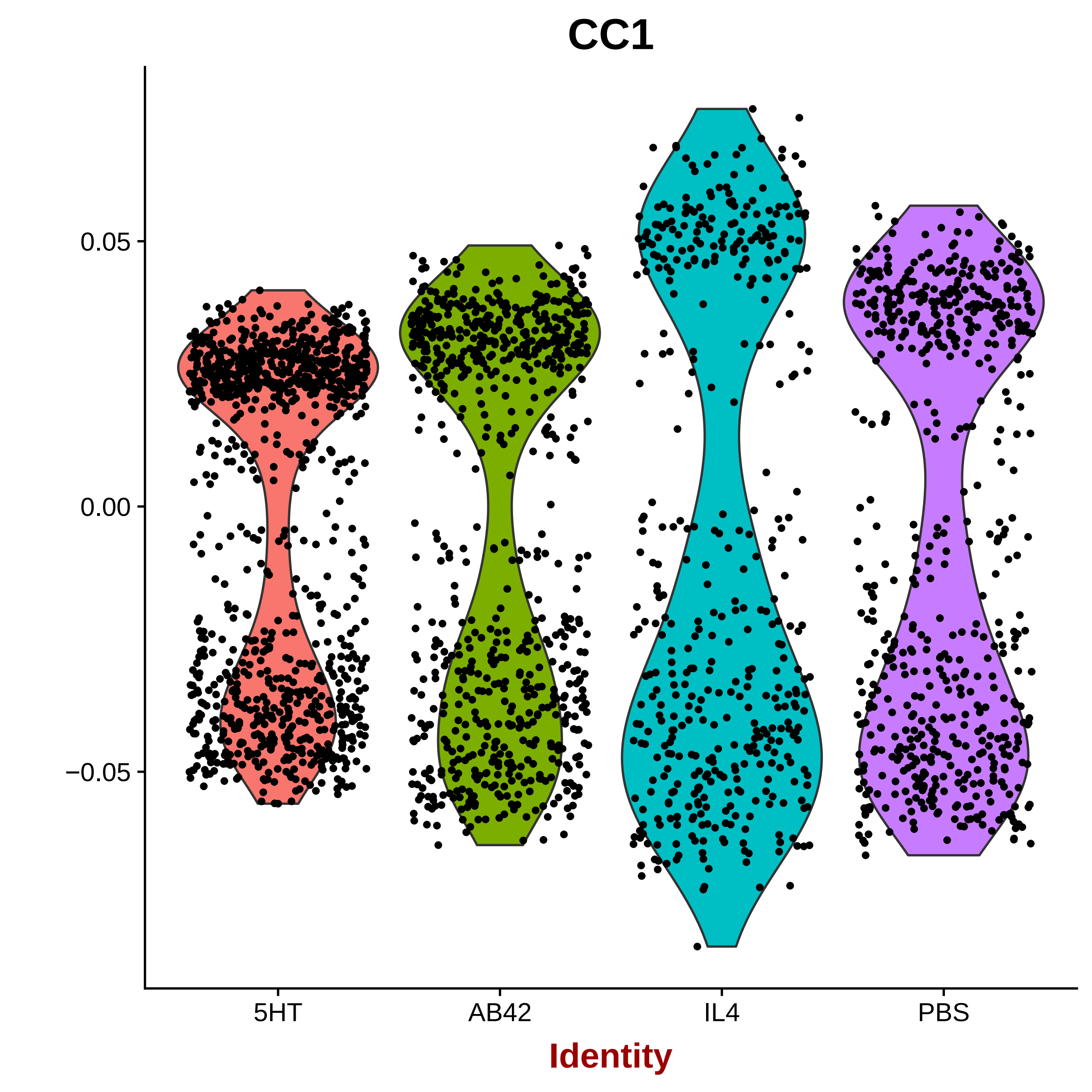
CC1
0.05
0.00
−0.05
5HT
AB42
IL4
PBS
Identity
